# Boosting Anion Transport Activity of Diamidocarbazoles by Electron Withdrawing Substituents

**DOI:** 10.3389/fchem.2021.690035

**Published:** 2021-05-20

**Authors:** Krystyna Maslowska-Jarzyna, Maria L. Korczak, Michał J. Chmielewski

**Affiliations:** Faculty of Chemistry, Biological and Chemical Research Centre, University of Warsaw, Warsaw, Poland

**Keywords:** anion transport, chloride transport, pH-switchable transport, liposomes, LUVs, lucigenin, carbazole

## Abstract

Artificial chloride transporters have been intensely investigated in view of their potential medicinal applications. Recently, we have established 1,8-diamidocarbazoles as a versatile platform for the development of active chloride carriers. In the present contribution, we investigate the influence of various electron-withdrawing substituents in positions 3 and 6 of the carbazole core on the chloride transport activity of these anionophores. Using lucigenin assay and large unilamellar vesicles as models, the 3,6-dicyano- and 3,6-dinitro- substituted receptors were found to be highly active and perfectly deliverable chloride transporters, with EC_50,270s_ value as low as 22 nM for the Cl^−^/NO_3_^−^ exchange. Mechanistic studies revealed that diamidocarbazoles form 1:1 complexes with chloride in lipid bilayers and facilitate chloride/nitrate exchange by carrier mechanism. Furthermore, owing to its increased acidity, the 3,6-dinitro- substituted receptor acts as a pH-switchable transporter, with physiologically relevant apparent pK_a_ of 6.4.

## Introduction

Small molecules able to transport polar species through lipid bilayers are highly appealing for their potential biological activities (de Riccardis et al., 2013). This is because in living cells the concentration gradients of cations, anions and other polar solutes need to be precisely controlled and molecules that effectively perturb these gradients are very likely to influence biological functions (Sperelakis and Sperelakis, 2012). Indeed, many ionophores show antiviral, antibacterial and antifungal activities and some of them have been shown to act against insects, pests, and parasites (Kevin II et al., 2009). Significant research efforts have also been directed toward the development of novel antitumor, anti-inflammatory, antioxidant and neuroprotective ionophores (Ding and Lind, 2009; Kevin II et al., 2009; Kaushik et al., 2018).

Among other polar solutes, chloride transport is gaining increasing interest in recent years due to the growing appreciation of its physiological importance (Pusch, 2007). Chloride is the most abundant anion in the extracellular fluids, where its concentration typically exceeds 100 mM (Davis et al., 2007). In contrast, the intracellular Cl^−^ concentration is one order of magnitude lower and varies considerably between different cell types. The resulting chloride concentration gradients are important physiological parameters and in living cells they are precisely controlled by specialized proteins: pumps, channels and carriers. The transmembrane chloride transport they provide is important for CO_2_ excretion and pH regulation, neural signaling and maintaining cell volume (Pusch, 2007). Mutations in genes coding for these proteins are responsible for severe diseases, such as cystic fibrosis (Sheppard et al., 1993; Welsh and Smith, 1993), Bartter syndrome (Simon et al., 1996; Simon et al., 1997), and Dent’s disease (Lloyd et al., 1996). One of the major driving forces behind the rapid development of artificial chloride transporters has been the idea that they might be used for treating these diseases (Gale et al., 2017; Davis et al., 2020). Apart from that, artificial chloride transporters have been shown to display also other interesting properties, such as anticancer and antibacterial activity (Elie et al., 2015; Saha et al., 2016).

**FIGURE 1:**
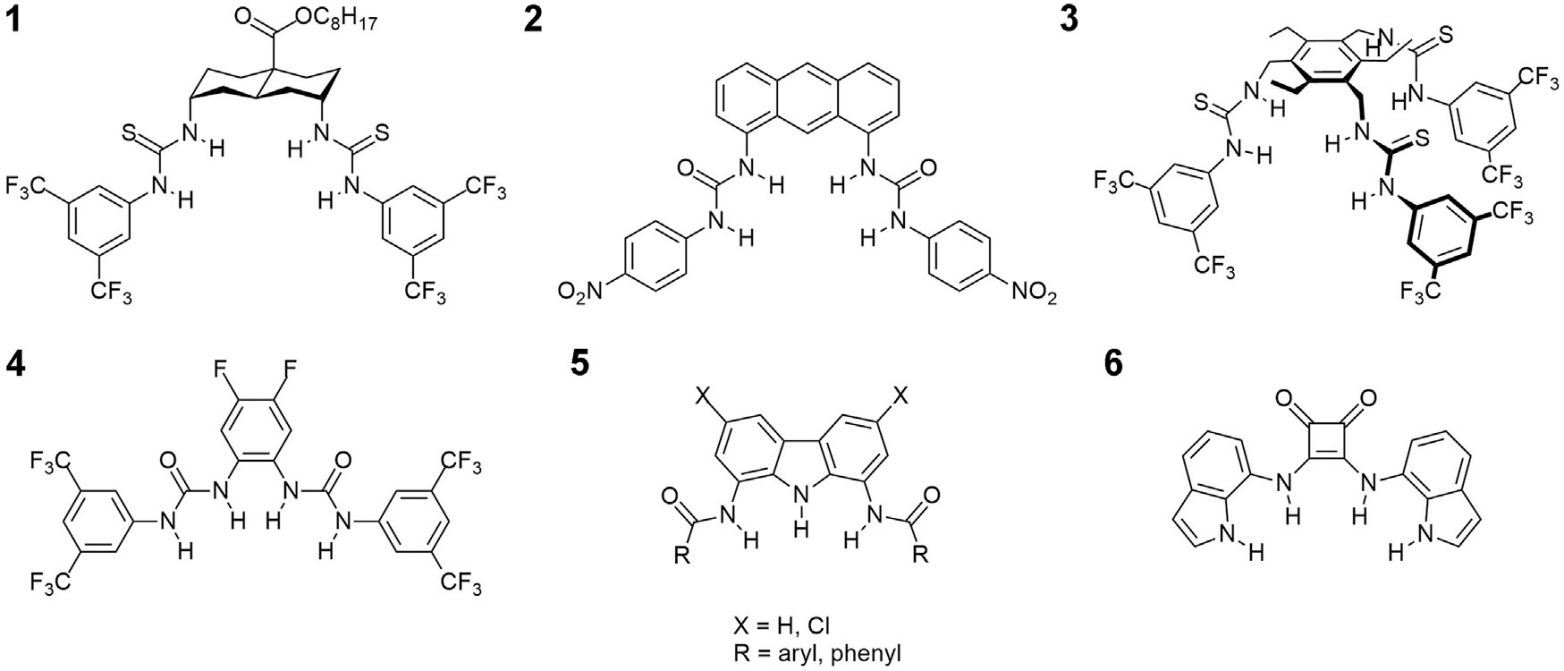
Examples of chloride transporters from literature: **1** - ref. (Valkenier et al., 2014); **2** - ref. (Dias et al., 2018b); **3** - ref. (Valkenier et al., 2015); **4** - ref. (Dias et al., 2018a), **5** - ref(Bąk et al., 2018); and **6** - ref. (Picci et al., 2020).

Not surprisingly therefore, a flood of reports on artificial chloride transporters have been published in recent years (Valkenier et al., 2014; Dias et al., 2018b; Valkenier et al., 2015; Dias et al., 2018a; Bąk et al., 2018; Picci et al., 2020; Pomorski et al., 2021). Most of these reports concern uncharged, hydrogen bonding receptors with several hydrogen bond donors arranged in a convergent manner and a relatively lipophilic outer surface (for selected examples see Figure 1). Owing to these features the receptors preferentially dissolve in the lipid bilayer and effectively extract anions from water through the formation of supramolecular complexes which are sufficiently lipophilic to permeate the membrane. Ureas, thioureas and squaramides are among the most frequently used anion binding motifs in this type of molecules (Li et al., 2016), and perfluorinated residues are often attached to improve transport rates (Busschaert et al., 2011).

Recently, we have shown that simple and easy to make 1,8-diamidocarbazoles **5** (Figure 1) might also be developed into very active chloride carriers. Their transport activity can be easily modulated by varying the length and degree of branching of the amide side chains as well as by appropriate substitution of the carbazole core. Ion selective electrode (ISE) assay employing large unilamellar POPC vesicles (POPC LUVs) gave EC_50_ values down to 0.1 μM for this initial set of carriers (Bąk et al., 2018).

**FIGURE 2:**
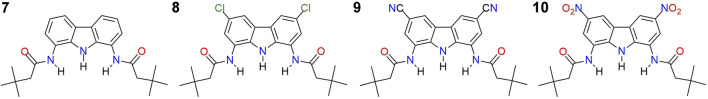
Diamidocarbazole transporters **7**–**10** investigated in the present study.

To further improve the chloride transport activity of 1,8-diamidocarbazoles, we decided to introduce strongly electron withdrawing substituents in positions 3 and 6 of the carbazole core. Similar strategy has been successfully used to boost the activity of various ion carriers (Moore et al., 2013; Peng et al., 2016; Yu et al., 2018) and channels (Zeng et al., 2020). Recently, we have developed the synthesis of 3,6-dicyano and 3,6-dinitro substituted diamidocarbazoles **9** and **10** (Figure 2) and found that their chloride affinity is 6 and 7 times higher, respectively, in comparison to the unsubstituted **7**.^1^ We found also that **9** and **10** are very prone to deprotonation. In this contribution, we study the chloride transport activity of the whole family of receptors **7**–**10** and investigate their mechanism of action, deliverability and pH dependent activity.

## Results and Discussion

### The Synthesis of Model Transporters

Model receptors **7**–**10** were synthesized according to the previously published procedures (Bąk and Chmielewski, 2014; Bąk et al., 2018)^1^. In brief, chlorination of carbazole in positions 3 and 6 followed by nitration in positions 1 and 8 gives the key precursor – 3,6-dichloro-1,8-dinitrocarbazole **11**. This compound can be then either gently hydrogenated to yield 1,8-diamino-3,6-dichlorocarbazole, the substrate for the synthesis of **8**, or heated with hydrazine hydrate in the presence of Pd/C to give 1,8-diaminocarbazole, the acylation of which gives **7**. Similar strategy has been applied for the synthesis of **9**: bromination of carbazole and subsequent Pd-catalyzed Br to CN exchange gave 3,6-dicyanocarbazole (Weselinski et al., 2014), which can be cleanly nitrated in positions 1 and 8. Selective reduction of the 3,6-dicyano-1,8-dinitrocarbazole, followed by acylation, yields **9**. Finally, the synthesis of **10** starts from the commercially available 1,3,6,8-tetranitrocarbazole, which is selectively reduced in positions 1 and 8 and acylated to give **10**.

### X-Ray Crystallographic Studies

**FIGURE 3:**
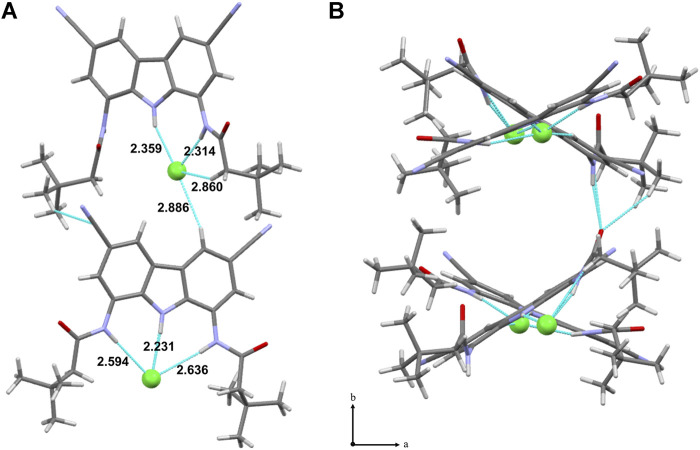
X-ray crystal structure of **9**×TBACl; the TBA^+^ counter cations were omitted for clarity. Hydrogen bonds between **9** and Cl^−^ are colored into light-blue. a) view along [010] direction; b) packing of **9**×TBACl, view along [001] direction.

An X-ray crystal structure was obtained for the complex of **9** with TBACl. There are two crystallographically independent chloride complexes in the unit cell, both showing chloride anion bound inside the carbazole cleft, as expected. However, in one of the complexes the anion is held by all three NH moieties of the receptor, whereas in the other–by only two (Figure 3A). This agrees with our previous presumptions that the chloride anion is too small to simultaneously form short hydrogen bonds with both amide groups of diamidocarbazoles. As a result, it either locates close to the middle of the binding cavity to form relatively long hydrogen bonds with both amides, or shifts to one side to maximize binding with only one amide. Both possibilities are nicely illustrated in the crystal structure of **9**×TBACl.

The aforementioned geometric mismatch between the binding cavity and the chloride anion is likely responsible for the relatively weak chloride affinity of diamidocarbazoles. As will be shown below, however, this rather weak binding is sufficient to ensure very fast chloride transport.

### Chloride Transport Studies

Previously, the anion transport properties of diamidocarbazoles were investigated using a standard ISE assay (Bąk et al., 2018). In this method, a solution of a receptor in a water-miscible solvent, such as DMSO, is added to a suspension of LUVs in water. The typically lipophilic receptor then partially precipitates, and partially partitions between the lipid bilayer and aqueous phase. Although operationally simple and medicinally relevant, this assay is not particularly well suited for structure-activity relationship studies because the intrinsic activity trends might be blurred by poor deliverability of some receptors due to solubility issues. Therefore, in the present study, we chose a fluorescent assay, in which the receptor is pre-incorporated into the lipid membrane during the LUVs’ preparation. In this method, the receptor-to-lipid ratio can be more precisely controlled.

On the other hand, even the most active transporters are not particularly attractive from medicinal chemistry point of view if they suffer from poor deliverability. This is likely to be the case for diamidocarbazoles, because they are very poorly soluble in water and in most organic solvents (except for aprotic dipolar solvents such as DMSO and DMF). To address this issue, we directly compared the activity of pre-incorporated and post-incorporated transporters.

**FIGURE 4:**
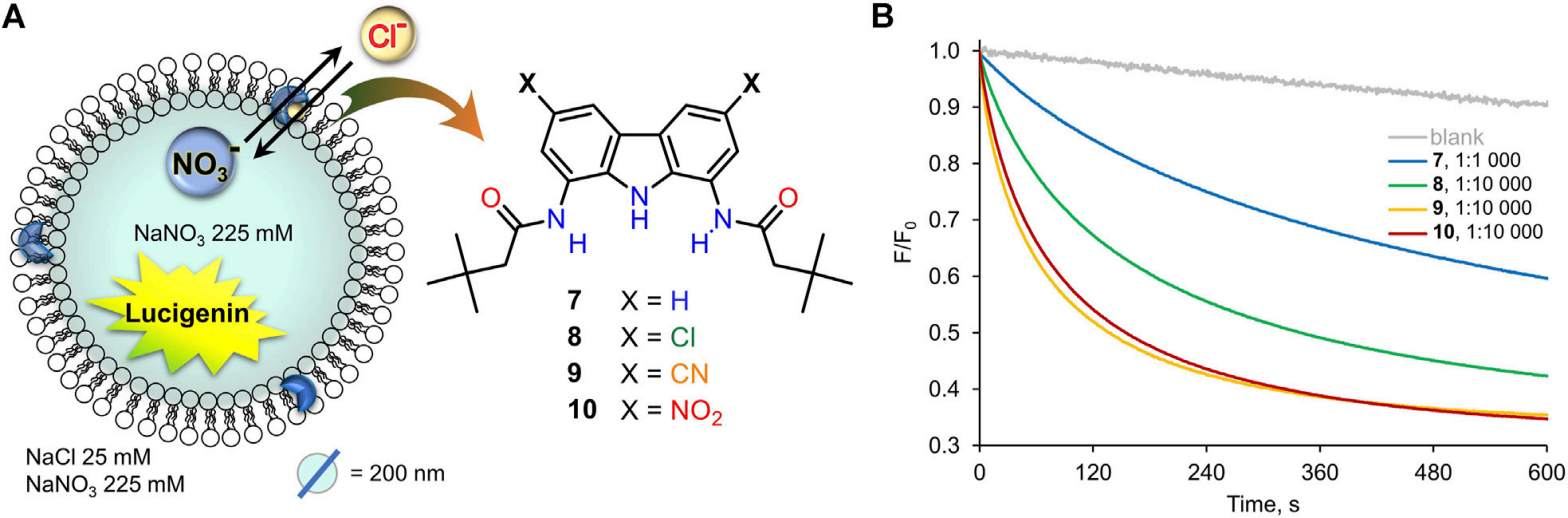
**(A)** Schematic representation of the lucigenin assay; **(B)** changes in the relative fluorescence *F/F*_*0*_ measured during the transport of Cl^−^ into 200 nm LUVs mediated by receptors **7**–**10** pre-incorporated in the membrane (**7**: 400 nM, **8**–**10**: 40 nM).

Thus, the chloride transport by **7**–**10** was initially studied using the very well-known lucigenin assay for Cl^−^/NO_3_^−^ exchange, schematically shown in Figure 4 (Valkenier et al., 2014). LUVs (mean diameter 200 nm) were grown from a lipid mixture composed of POPC and cholesterol (in 7:3 ratio) with the appropriate transporter at a given concentration, in the presence of halide-sensitive dye, lucigenin (0.8 mM), and NaNO_3_ (250 mM). Unencapsulated lucigenin was separated from vesicles by gel filtration. Such liposomes were then suspended in aqueous NaNO_3_ (225 mM), diluted to 0.4 mM and transferred to a spectrofluorometer. The experiment was commenced by the addition of NaCl (1M in 225 mM NaNO_3_) to a final concentration of 25 mM. The addition of NaCl resulted in an immediate drop of fluorescence intensity due to quenching of the residual unencapsulated lucigenin, followed by a gradual decrease of fluorescence due to the transport-mediated quenching of the internal lucigenin by chlorides (Figure 4A).

All the carbazole-based receptors **7**–**10** were found to be active chloride transporters under these conditions (Figure 4B). However, the electron-deficient **8**–**10** promoted much faster transport than the unsubstituted **7** (note that in Figure 4B the concentrations of **8**–**10** are 10 times lower than **7**). The 1:10,000 receptor-to-lipid ratio corresponds to just 0.01 mol% concentration of the receptor in the bilayer and 0.04 μM in the whole vesicle suspension. Even when present in so tiny amounts, the most active carriers **9** and **10** almost completely equilibrate chloride concentrations on both sides of the membrane in 10 min.

The transport rates were quantified by fitting the inverse of the normalized fluorescence traces *F*_*0*_ */F* to a single exponential function to obtain approximate half-lives *t*_*1/2*_ and to a double exponential function to obtain initial rates *I*. Results are summarized in Table 1.

**TABLE 1:** Binding and transport data for receptors **7**–**10**.

	K_a_ [M^−1^]^a)^	*t*_*1/2*__pre_ [s]^b)^	*I*_pre_ [s^−1^]^c)^	*I*_post_ [s^−1^]^c)^	σ_p_^d)^	D^e)^	clogP^f)^
**7**	47.8	n.d. (648*)	n.d. (17*)	n.d	0.00	n.d	5.02
**8**	158.5	276	56	44	+0.23	0.79	5.85
**9**	309.0	140	126	146	+0.66	1.16	4.10
**10**	346.7	163	115	128	+0.78	1.11	4.61

n.d. = not determined; *- parameters obtained for 1:1 000 transporter:lipid ratio. ^a)^ Obtained through ^1^H NMR titrations with TBACl in DMSO +0.5% H_2_O at 298 K (Bąk et al., 2018;)^1^. ^b)^ Half-life times *t*_*1/2*_ obtained from fitting of *F*_*0*_ */F* (0–600 s) traces to a single exponential function for pre-incorporated **7**–**10** at transporter-to-lipid ratio 1:10,000. ^c)^ Initial rate *I* obtained from fitting of *F*_*0*_ */F* (0–600 s) traces to a double exponential function for pre-incorporated (*I*_pre_) or post-incorporated (*I*_post_) **7**–**10**; transporter:lipid = 1:10,000. ^d)^ Hammett parameters for substituents in positions 3 and 6. ^e)^ Deliverability D calculated as the ratio of initial transport rates derived from experiments with post- and pre-incorporated transporter, transporter-to-lipid ratio 1:10,000. ^f)^ Calculated logP values predicted using ALOGPS 2.1 software; *p* is the partition coefficient of the receptor between n-octanol and water.

The most noticeable result is a huge, more than tenfold, increase in activity upon introduction of moderately electron withdrawing chlorine substituents in positions 3 and 6 of the carbazole core. This effect has already been noted in our previous study using ISE assay, but the results presented here prove that it is related to the intrinsic activities of **7** and **8**. Receptor **8** has 3 times higher chloride affinity than **7** and also higher clogP value, so we presume that the combined effect of these two changes is responsible for such a strong impact on ionophoric activity.

Further increase in electron withdrawing strength from Cl to CN increases the chloride transport rate more than twofold, in line with the *ca.* twofold increase in anion affinity. It is well known, however, that many other factors have significant impact on the observed transport rates, so this corelation might be a coincidence. In fact, the CN-substituted receptor **9** is also much less lipophilic than **8**, with clogP = 4.10 vs. 5.85 for **8**.

Finally, the most electron withdrawing NO_2_ substituents slightly decrease the transport activity of **10**, despite increased chloride affinity and higher lipophilicity in comparison to **9**. As will be shown below, this is probably due to partial deprotonation of **10** under these conditions.

**FIGURE 5:**
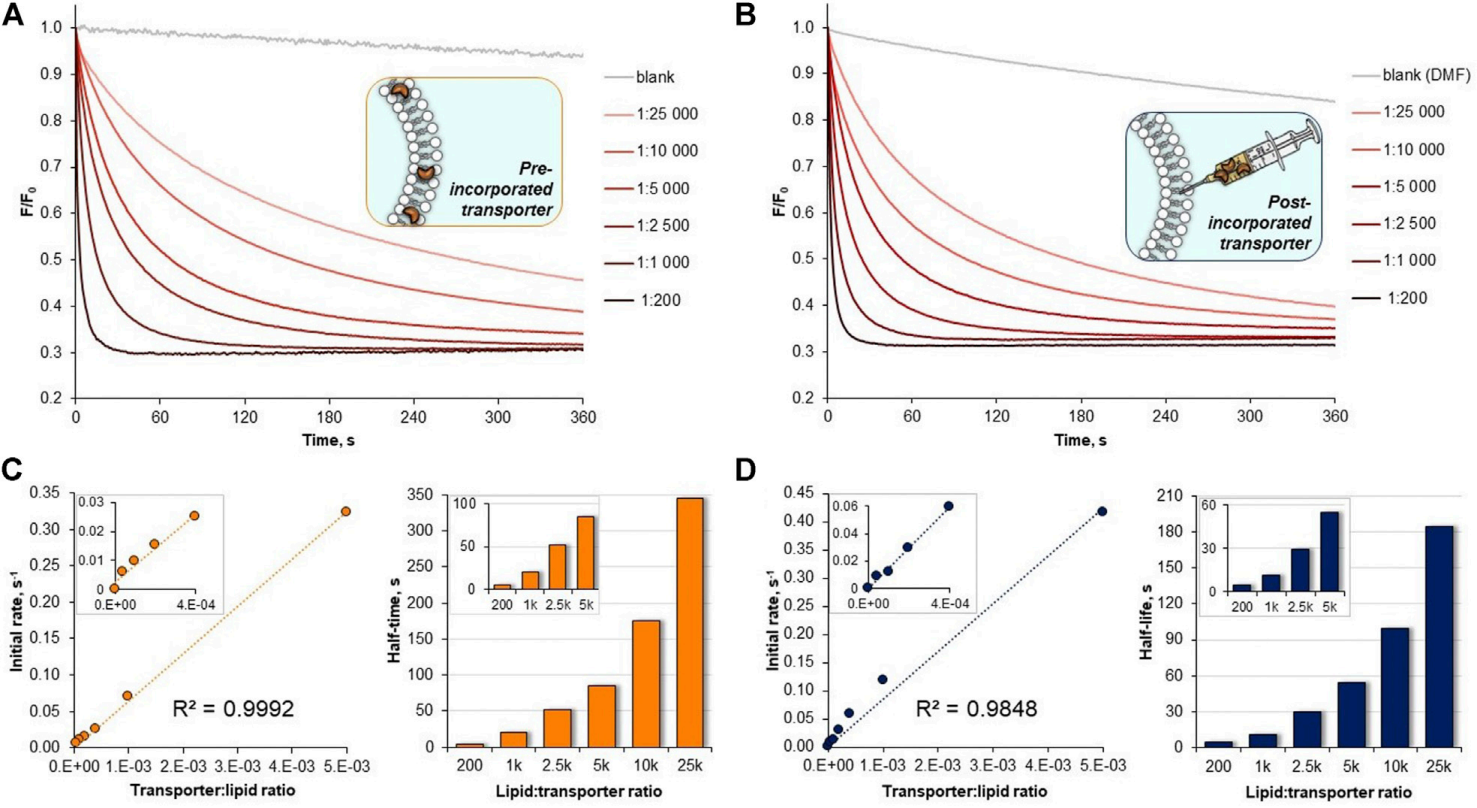
**(A)** Raw data from the chloride transport studies by **10 (A)** pre-incorporated in the membrane, **(B)** post-incorporated in the membrane. Plots of initial rates *I vs* transporter:lipid ratio for **10** and corresponding results from half-life times *t*_*1/2*_ calculations: **(C)** pre-incorporated anionophore, **(D)** anionophore added externally as a solution in DMF.

Dose-activity studies were performed for **10** to allow for Hill analysis and calculation of EC_50,270s_ value (Figure 5A). In our assay, this value refers to the concentration of pre-incorporated transporter required to rise the intravesicular chloride concentration to 50% of the initial gradient during 270 s. Using *F*_*0*_ */F* as a measure of chloride concentration inside the vesicles, we plotted the values recorded at 270 s against the concentration of transporter **10** in the membrane and fitted the data with the Hill equation (Supplementary Figure S36). Using this procedure, EC_50,270s_ of 22.4 nM (0.0056 mol% with respect to lipids) was obtained. Although it is difficult to compare literature values obtained under different conditions and using different assays, this level of activity certainly ranks **10** among the most active artificial transporters known to date.

Hill analysis also allows for the Hill coefficient to be determined, which indicates the number of receptor molecules in the active complex. The calculated value of 1.08 ± 0.07 indicates that **10** acts as a mobile carrier and not as a channel. This is in agreement with the linear correlation between the initial transport rates and the transporter concentration, presented in Figure 5C.

To shed more light on the mechanism of anion transport by **7**–**10**, additional control experiments were performed in which the relatively lipophilic NO_3_^−^ anions were replaced by highly hydrophilic SO_4_^2−^ in both internal and external solutions (Grauwels et al., 2019). No significant anion transport was detected under these conditions (ESI, Supplementary Figure S4), which means that the Cl^−^/NO_3_^−^ exchange is the most likely mechanism for the transport activity displayed by **7**–**10**. Similar results were previously obtained for other diamidocarbazoles and di (thioamido)carbazoles (Bąk et al., 2018; Pomorski et al., 2021).

**FIGURE 6:**
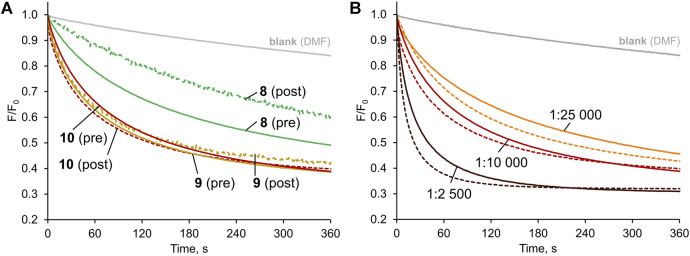
Chloride transport with the anionophores either pre-incorporated in the membrane (solid lines) or added externally as a solution in DMF to vesicles without anionophore (dashed lines), mediated by: **(A) 8–10** (1:10,000 transporter:lipids ratio); **(B) 10** at various transporter:lipids ratios.

Having established diamidocarbazoles **8**–**10** as very active chloride transporters, we next sought to investigate how readily they could be delivered into lipid membranes by post-assembly addition. As mentioned before, this is an important aspect of practical effectiveness and sometimes even the most active synthetic transporters are unable to pass this test satisfactorily (Valkenier et al., 2014; Mora et al., 2016). To investigate deliverability, LUVs were prepared in a manner analogous to that previously described, except that no anionophore was added to the initial lipid mixture. Instead, 5 μL of concentrated solution of receptor in DMF (Dias et al., 2018b) was added to the rapidly stirred suspension of preformed vesicles. After 3 min of stirring, 1M NaCl was added and the decay in fluorescence was measured as before (Figure 6).

Visually, the traces for pre-incorporated and post-incorporated **9** and **10** are close to each other, meaning that the deliverability of these receptors is very good. Much more significant difference was observed between the respective traces for **8**, while **7** was not investigated due to the greatly inferior activity.

To quantify the effect, the deliverability factor D was calculated for **8**–**10** by dividing the initial rate of transport for post-incorporated receptor by the value obtained for pre-incorporated anionophore. In theory, for perfectly deliverable transporters, D should be equal to 1. However, the actual concentration of lipids is always lower than the nominal value due to losses during vesicle preparation, and therefore in experiments with post-incorporated receptors the real transporter-to-lipid ratio is always higher than intended (Bąk et al., 2018). As a result, D is sometimes higher than 1 and this was indeed observed for **9** and **10**. The same effect can be observed by comparing the *F/F*_*0*_ traces for post- and pre-incorporated **10** at various receptor-to-lipid ratios–the post-incorporated ionophore consistently shows higher apparent activity (Figure 6B). On the contrary, however, **8** displayed inferior activity when added externally, indicating that this receptor is not perfectly deliverable (D = 0.79). Even in this least favourable case, however, the deliverability issue is not so severe as to rule out potential applications of this anionophore.

### pH-Dependent Transport Studies

pH-switchable anionophores are particularly attractive for their potential medical applications. Such compounds may be used to specifically target chloride transport in acidic cells and organelles, and to couple chloride transport with proton gradients. Since cancer cells are generally associated with lower pH values than healthy cells, it would be very beneficial if synthetic transporters could effectively switch ON in acidic environment and OFF at neutral pH. To date, however, there are only few reports on pH-dependent transporters (Busschaert et al., 2014; Elmes et al., 2015; Howe et al., 2016; Saha et al., 2019; Wang et al., 2021) and highly active carriers that possess very specific pH-regulated ON/OFF function are still unavailable.

The 3,6-dinitro substituted diamidocarbazole **10** is significantly more acidic than other diamidocarbazoles, so we were curious if it may function as pH-switchable carrier. To address this question experimentally, we decided to use the lucigenin method. Lucigenin fluorescence is not significantly affected by pH up to 11 (Bąk et al., 2021), so it seemed to be well suited for this purpose. To the best of our knowledge, however, related studies of pH-dependent chloride transport using lucigenin assay have not been reported in the literature, despite its ease and availability.

**FIGURE 7:**
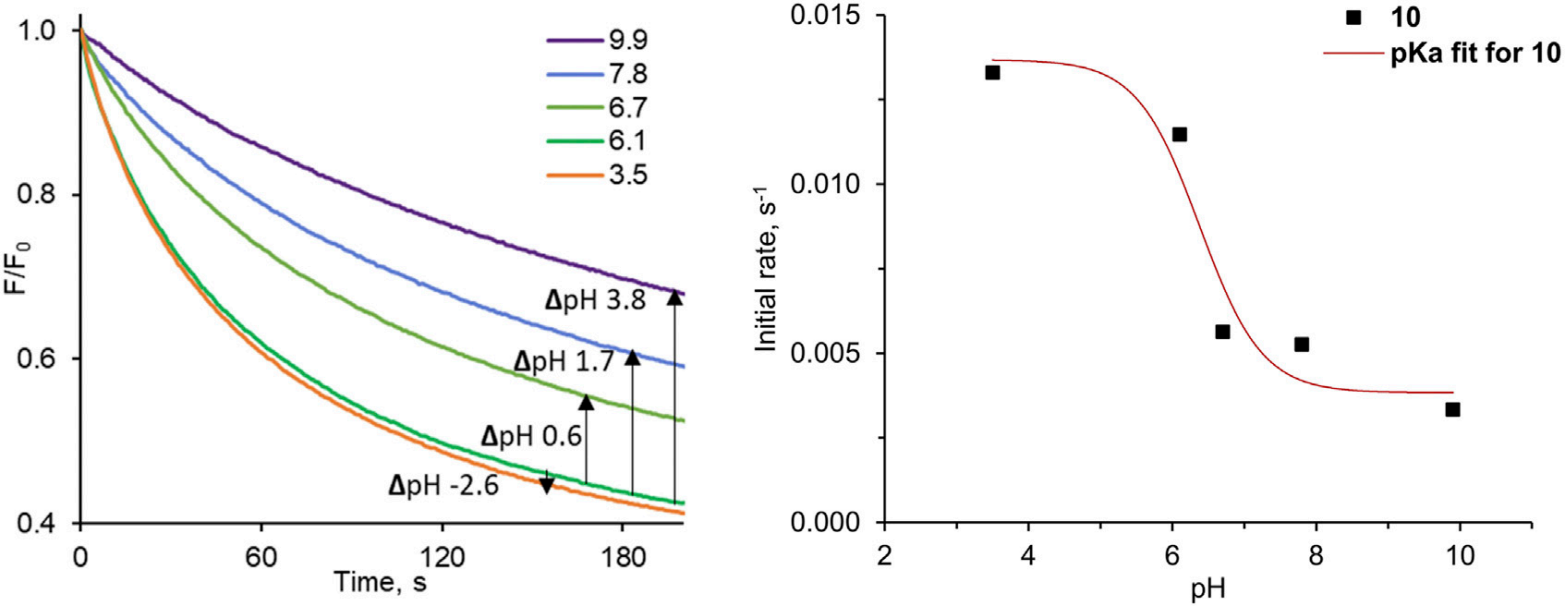
Relative fluorescence *F/F*_*0*_ traces measured as a function of pH for the pH-dependent Cl^−^ transport into 200 nm LUVs with pre-incorporated **10** at 1:10,000 transporter:lipids ratio.

The results of our pH-dependent transport experiments are shown in Figure 7. Quantification of the transport rates was accomplished as before, by fitting each *F*_*0*_ */F* curve (0–600 s) to a double exponential decay function in order to determine initial rates *I*. As expected, the transport activity of **10** is indeed the highest at acidic pH, and drops significantly at pH close to neutral (Figure 7). The particularly striking decrease in activity was observed between pH 6.1 and 6.7, where this small pH change reduces transport rate ca. 2-fold. The plot of the initial transport rates vs. pH was used to calculate the apparent pK_a_ of **10** in the membrane environment, according to the method developed by Gale and co-workers (see ESI for details) (Busschaert et al., 2014). The obtained value of apparent pK_a_ = 6.4 is very close to physiological pH.

## Conclusion

Simple diamidocarbazoles with highly electron withdrawing nitro- and cyano-substituents in the carbazole core have been established as extremely active and perfectly deliverable chloride transporters in large unilamellar vesicles. Compound **10** gave a very low EC_50, 270s_ value of 22.4 nM in the chloride/nitrate assay, which is particularly impressive considering its simple structure and easy availability. Mechanistic studies revealed that diamidocarbazoles act as chloride/nitrate exchangers and form 1:1 complexes with chloride while carrying it through lipid bilayers. The 3,6-dinitro substituted receptor **10** is much more acidic than other diamidocarbazoles and hence its activity is significantly dependent on pH. The receptor is most active in mildly acidic medium and loses much of its activity at pH close to physiological. Taken together, all these findings make **10** a very promising lead compound for further development of chloride transporters for medicinal applications.

## Materials and Methods

### Abbreviations

ISE: ion-selective electrode
LUV: large unilamellar vesicle
POPC: 1-palmitoyl-2-oleoyl-*sn*-glycero-3-phosphocholine;
DMSO: dimethyl sulfoxide.

### Materials

All solvents and reagents were commercially available and used as received unless otherwise stated.

Water was taken from Milli-Q purification system.

#### Chempur

Methanol (116219904, >99.8%).

#### GE Healthcare

Sephadex G-50 superfine (17004101).

#### Sigma-Aldrich

Aluminum oxide (199443, activated, basic), *N*,*N′*-dimethyl-9,9′-biacridinium dinitrate (lucigenin, M8010), chloroform (for HPLC, stab. with amylene, 34,854-1L-M, ≥99.8%), cholesterol (C8667, >99.5%), nitric acid (84,378, puriss. p. a., 65–67%), 2-oleoyl-1-palmitoyl-*sn*-glycero-3-phosphocholine (POPC, 850457C, >99%, Avanti PC and LPC), sodium hydroxide solution (2 N, Titripur, 1091361000), sodium nitrate (221341, ≥99.0%).

#### VWR

Dimethylforamide (DMF, for HPLC, 83635320, >99.90%).

### Instruments and Methods

#### Weighing

Mettler Toledo Excellence XA105DU analytical balance was used for weighing all the samples.

#### Fluorescence spectroscopy

Fluorescence spectra were acquired using Hitachi F-7000 spectrophotometer equipped with Peltier temperature controller. Screw-capped SUPRASIL quartz fluorescence cuvettes (optical path length: 10 mm) were used.

#### Vortexing

IKA VORTEX 4 basic, model V 4 B S000 was used for vortexing during LUVs preparation.

#### Extrusion

AVESTIN LiposoFast-Basic with polycarbonate membranes with pore sizes of 200 nm was used for extrusion during LUVs preparation.

### Synthesis of Receptors 7–10

Transporters **7–10** were obtained as described previously. (Bąk et al., 2018; Bąk and Chmielewski, 2014)^1^.

### General Procedure for Measuring the Transport of Chlorides in LUVs Using Pre-incorporated Receptors

The general procedure is a slightly modified version of the chloride transport protocol published previously (Valkenier et al., 2014). In a 5 ml round bottom flask the mixture of 420 µL of 10 mM POPC and 180 µL of 10 mM cholesterol (7:3 M ratio) was prepared in chloroform which was previously deacidified by passing through a pad of activated basic alumina. 10 µL of a solution of an appropriate anionophore **7**–**10** (0.024 mM, 0.06 mM, 0.24 mM, or 3 mM in CHCl_3_:MeOH 1:1 v/v) were added to the lipids solution at 0–0.5 mol% ratio relative to the total amount of lipids. Neither the receptor solution nor pure CHCl_3_:MeOH 1:1 v/v were added when the blank was measured (0 mol%). The organic solvents were evaporated on a rotary evaporator and the residue was dried under high vacuum for 1 h. The lipid film was hydrated with 0.500 ml of aqueous solution containing lucigenin (0.8 mM) and NaNO_3_ (225 mM) or Na_2_SO_4_ (112 mM), sonicated for 30 s and vortexed for 1 h resulting in vesicle formation. The multilamellar vesicles were broken down into unilamellar vesicles by 10 freeze-thawing cycles, diluted to 1 ml by the addition of NaNO_3_ (225 mM) or Na_2_SO_4_ (112 mM), and extruded 29 times through a polycarbonate membrane (200 nm pore size) to give homogeneous large unilamellar vesicles. Unencapsulated lucigenin was removed by passing the mixture through a column with Sephadex 50G (ca. 2 g, superfine) using an aqueous NaNO_3_ (225 mM) or Na_2_SO_4_ (112 mM) as eluent. The collected vesicles were diluted with NaNO_3_ (225 mM) or Na_2_SO_4_ (112 mM) to 15 ml (lipid concentration ≈0.4 mM). 3 ml of the vesicle suspension was placed in a quartz cuvette with a small stirring bar and its fluorescence was measured as a function of time (excitation: 455 nm, emission: 505 nm). Aqueous NaCl (75 μL, 1.0 M in 225 mM NaNO_3_ or 112 mM Na_2_SO_4_, to give an overall external chloride concentration of 25 mM) was added 30 s after the start of fluorescence measurement. The fluorescence intensity was measured for 15 min. For each run the initial plateau (before the addition of chloride) and the vertical drop (the first 0.5–2 s after chloride addition) due to quenching of residual external lucigenin were removed. Next, the data were normalized: all fluorescence values (F) were divided by the maximum fluorescence value ($F_{0}$).

### General Procedure for Measuring the Transport of Chlorides in LUVs Induced by Externally Added Transporter

LUVs were prepared in the same manner as described in Section 4.5, except that the anionophore was not added to the initial lipid mixture. After size exclusion chromatography, the collected vesicles were diluted with aqueous NaNO_3_ (225 mM) to 15 ml (lipid concentration ≈0.4 mM) and 3 ml of this was transferred to a cuvette with a small stirring bar. Solutions of anionophores **7**–**10** were prepared in DMF (Dias et al., 2018b) at several concentrations: 1200 μM, 240 μM, 96 μM, 48 μM, 24 μM, 9.6 µM, 4.8 µM, 2.4 µM, 0.96 µM, 0.48 µM. Anionophore solution (5 µL) was then added to the vesicle suspension in the cuvette, employing a 25 µL syringe with rapid plunger action and with the tip of the syringe needle positioned just above the stir bar. Assuming the lipid concentration of 0.4 nM, this would give a transporter to lipid ratio of 1:200, 1:1 000, 1:2 500, 1:5 000, 1:10,000, 1:25,000, 1:50,000, 1:100,000, 1:250,000 or 1:500,000 respectively. Aqueous NaCl (75 μL, 1.0 M in 225 mM NaNO_3_, to give an overall external chloride concentration of 25 mM) was added 3 min after the anionophore addition and 30 s after the start of fluorescence measurements. The fluorescence intensity was measured for 15 min (excitation: 455 nm, emission: 505 nm). Pure DMF (5 µL) was added followed by the addition of aqueous NaCl (75 μL, 1.0 M in 225 mM NaNO_3_) to measure blank. The plateau (before addition of anions) and the vertical drop (the first 0.5–2 s after the addition of anions) due to quenching of residual external lucigenin were removed. The data are normalized: all fluorescence values (F) were divided by the fluorescence value measured before the addition of anion ($F_{0}$).

### Investigation of pH-dependent Chloride Transport

LUVs were prepared in the same manner as described in Section 4.5, except that transporter **10** (0.0015 M in CHCl_3_:MeOH 1:1 v/v) was diluted to a concentration of 60 µM and added to the lipids solution at 0.1 mol% (1:10,000) ratio relative to the total amount of lipids. After size exclusion chromatography, the collected vesicles were diluted with aqueous NaNO_3_ (225 mM) to 15 ml (lipid concentration ≈0.4 mM) and 3 ml of this was transferred to a cuvette with a small stirring bar. Calculated amounts of diluted solutions of NaOH or HNO_3_ were added to the liposomes suspension to achieve an appropriative pH. Additionally, final pH in each cuvette was measured using a pH-meter. Aqueous NaCl (75 μL, 1.0 M in 225 mM NaNO_3_, to give an overall external chloride concentration of 25 mM Cl^−^) was added 30 s after the start of fluorescence measurements. For each run the initial plateau (before the addition of chloride) and the vertical drop (the first 0.5–2 s after chloride addition) due to quenching of residual external lucigenin were removed. The data are normalized: all fluorescence values (F) were divided by the maximum fluorescence value ($F_{0}$).

### Quantification of Transport Rates

As described in previous publications (Elie et al., 2015), the inverse of the normalized fluorescence trace (*F*_*0*_ */F*) is directly proportional to the concentration of chloride inside the vesicles (according to the Stern-Volmer equation). Thus *F*_*0*_ */F* was used in the quantification of transport rates.

Half-life time, *t*_*1/2*_: determined by fitting the *F*_*0*_ */F* curve (0–600 s) to a single exponential decay function:

$$F_{0}/F\mathrm{=y-a}\cdot \exp\left(\mathrm{-bt}\right),$$

Obtaining a value for the fit parameter *b* allows for the half-life *t*_*1/2*_ to be calculated:

$$t_{\mathrm{1/2}}=\ln\left(2\right)/b,$$

Initial rate, *I*: determined by fitting of the *F*_*0*_ */F* curve (0–600 s) to a double exponential decay function:

$$F_{0}/F\mathrm{=y-a}\cdot \exp\left(\mathrm{-bt}\right)\mathrm{-c}\cdot \exp\left(\mathrm{-dt}\right),$$

Differentiating at *t* = 0 allows for the initial rate *I* to be calculated:

I = (a⋅b)+ (c⋅d),

Using *F*_*0*_ */F* values at 270 s as a measure of chloride concentration inside the vesicles at that time, these values were plotted against the corresponding concentration of transporter used for different loadings of transporter. The data were fitted with the Hill1 equation in Origin 2021 (Supplementary Figures S36–S37).

### X-Ray Measurement

For crystallizations of **9**×Cl^−^ complex, dichloroethane solution of **9** with excess of TBACl salt was mixed with pentane, placed into vials and NMR tubes, capped and left standing. Red crystalline material precipitated in NMR tube. Suitable crystal was chosen for the X-ray measurement.

Diffraction data were collected on the Agilent Technologies SuperNova Dual Source with the MoKα radiation (*λ* = 0.71073 Å). The lattice parameters were obtained by least-squares fit to the optimized setting angles of the reflections collected by using the CrysAlis CCD software (Oxford Diffraction, 2008). Data were reduced using the CrysAlis RED program (Oxford Diffraction, 2008). The Gaussian numerical absorption correction using a multifaceted crystal model implemented in SCALE3 ABSPACK scaling algorithm, was applied (Oxford Diffraction, 2008). Using Olex2 (Dolomanov et al., 2009), the structure was solved with the ShelXT (Sheldrick, 2015b) structure solution program using Intrinsic Phasing and refined with the ShelXl (Sheldrick, 2015a) refinement package using Least Squares minimization. All H-atoms were positioned geometrically. The crystallographic data are summarized in Supplementary Table S1. The values of bond lengths and valence angles are given in Supplementary Tables S2–S3.

Data Availability Statement: Crystallographic data for the structure in this paper have been deposited with the Cambridge Crystallographic Data Center as supplementary publication number CCDC 2074654. Copies of the data can be obtained, free of charge, on application to CCDC [e-mail: deposit@ccdc.cam.ac.uk, website: www.ccdc.cam.ac.uk].

## Data Availability

The datasets presented in this study can be found in online repositories. The names of the repository/repositories and accession number(s) can be found in the article/Supplementary Material.

## Footnotes

1 Maslowska-Jarzyna, K., Korczak, M. L., Wagner, J., and Chmielewski, M. J. (2021). Carbazole-based Colorimetric Anion Sensors. Molecules.submitted.
