# Supplementary material for: Boosting Anion Transport Activity of Diamidocarbazoles by Electron Withdrawing Substituents
Source: Front Chem. 2021 May 20;9:690035. doi: 10.3389/fchem.2021.690035 (PMC8172623; doi:10.3389/fchem.2021.690035)
Supplement: Supplementary file 1 [file DataSheet1.PDF]

Supplementary Material

Table of contents

1. Anion transport in LUVs by receptors preincorporated in membrane 2
2. Mechanistic studies 3
3. Anion transport in LUVs by post-incorporated receptors 4
4. Data fitting of results from transporter preincorporation method 7
5. Data fitting of results from externally added transporter method 12
6. Deliverability studies.. 19
7. Hill analysis for receptor **10** 20
8. Quantification of pH-dependent chloride transport rates 21
9. Crystal data and structure refinement.. 22

1. Anion transport in LUVs by receptors preincorporated in membrane

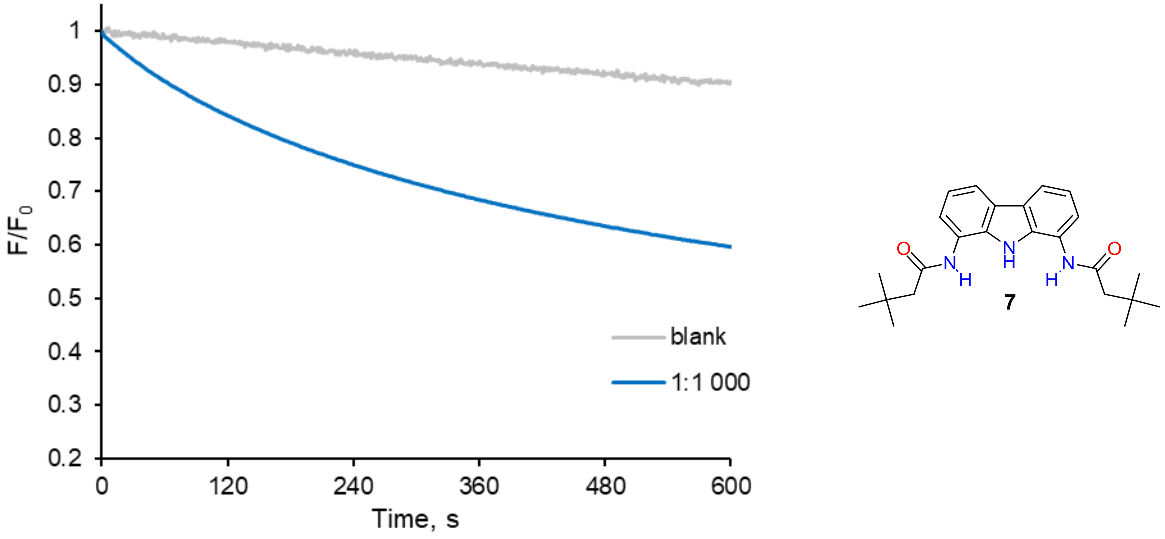

Supplementary Figure 1. Relative fluorescence F/F_0 for the transport of Cl^- into 200 nm LUVs by **7** preincorporated in the membrane at 1:1 000 transporter:lipid ratio.

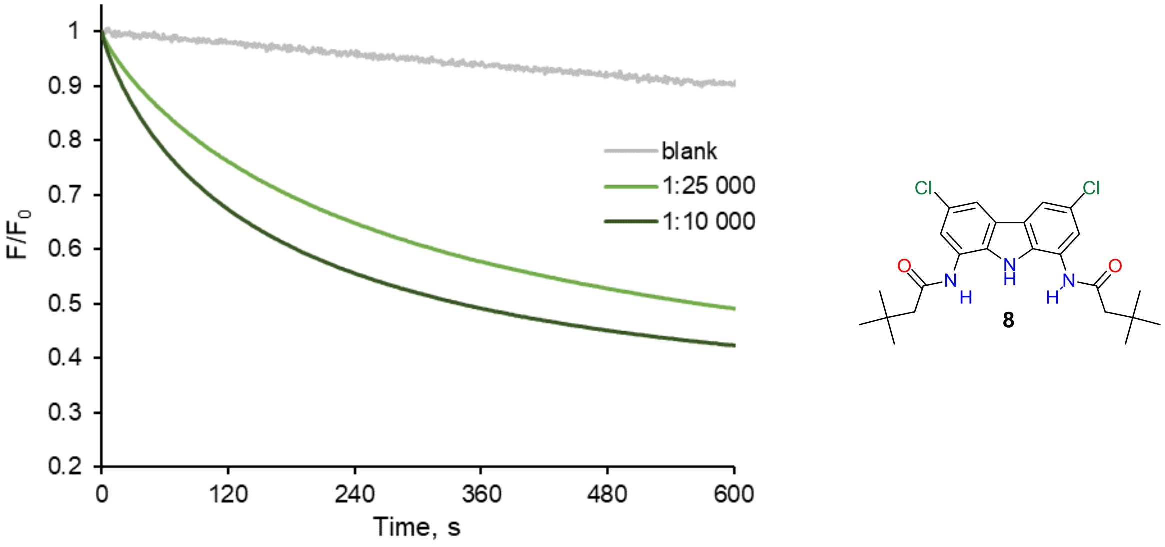

Supplementary Figure 2. Relative fluorescence F/F_0 for the transport of Cl^- into 200 nm LUVs by **8** preincorporated in the membrane at 1:10 000 and 1:25 000 transporter:lipid ratios.

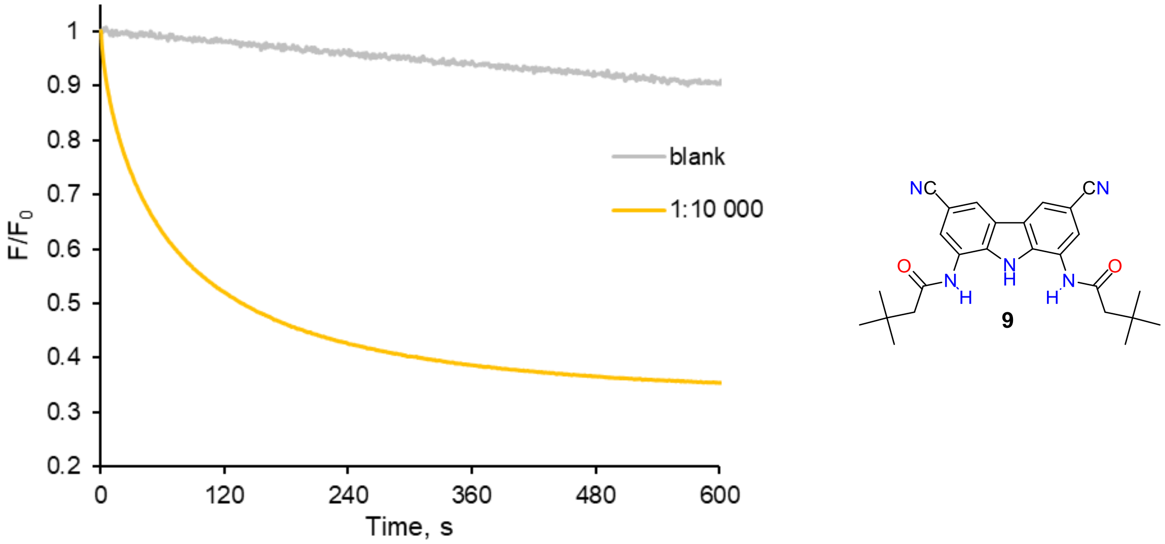

Supplementary Figure 3. Relative fluorescence F/F_0 for the transport of Cl^- into 200 nm LUVs by **9** preincorporated in the membrane at 1:10 000 transporter:lipid ratio.

2. Mechanistic studies

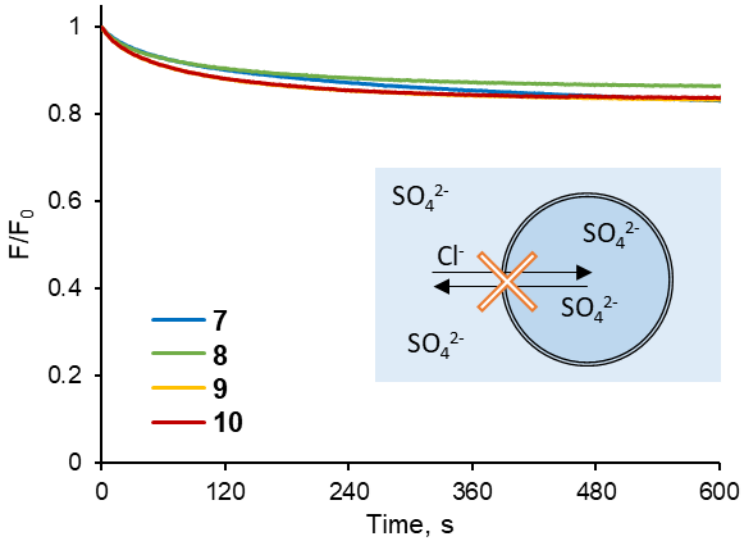

Supplementary Figure 4. Relative fluorescence F/F_0 for the transport of Cl^- into 200 nm LUVs by transporters **7-10** preincorporated in the membrane at 1:10 000 transporter:lipid ratio.

3. Anion transport in LUVs by post-incorporated receptors

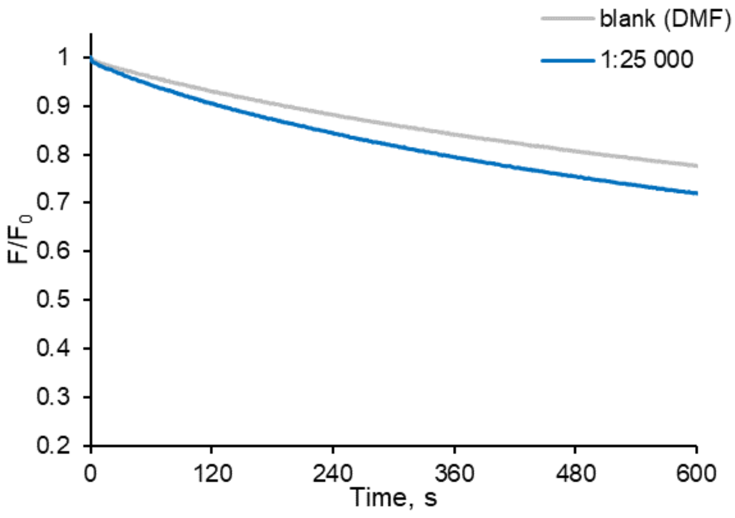

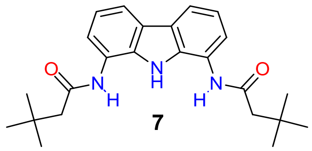

7

Supplementary Figure 5. Relative fluorescence *F/F₀* for the transport of Cl^- into 200 nm LUVs by **7** added externally as solution in DMF at 1:25 000 transporter:lipids ratio.

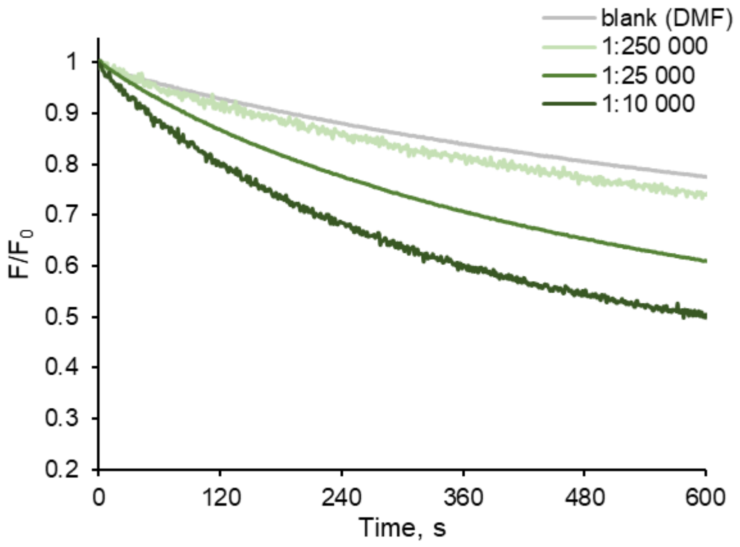

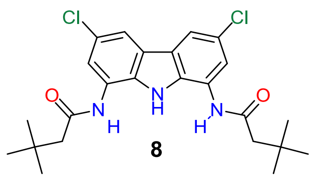

8

Supplementary Figure 6. Relative fluorescence *F/F₀* for the transport of Cl^- into 200 nm LUVs by **8** added externally as solution in DMF at various transporter:lipids ratios.

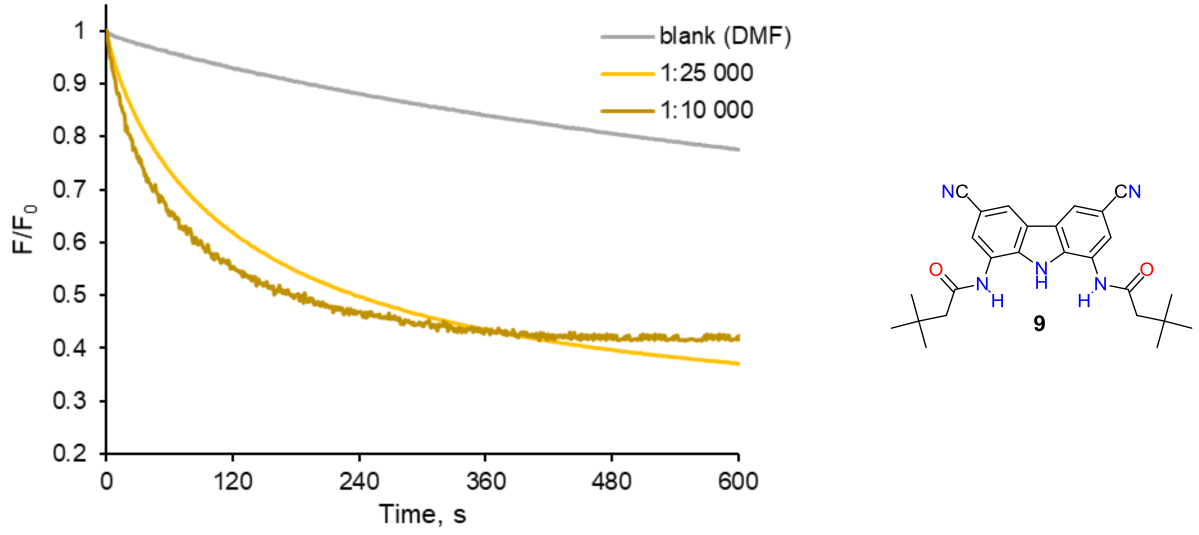

Supplementary Figure 7. Relative fluorescence F/F_0 for the transport of Cl^- into 200 nm LUVs by **9** added externally as solution in DMF at various transporter:lipids ratios.

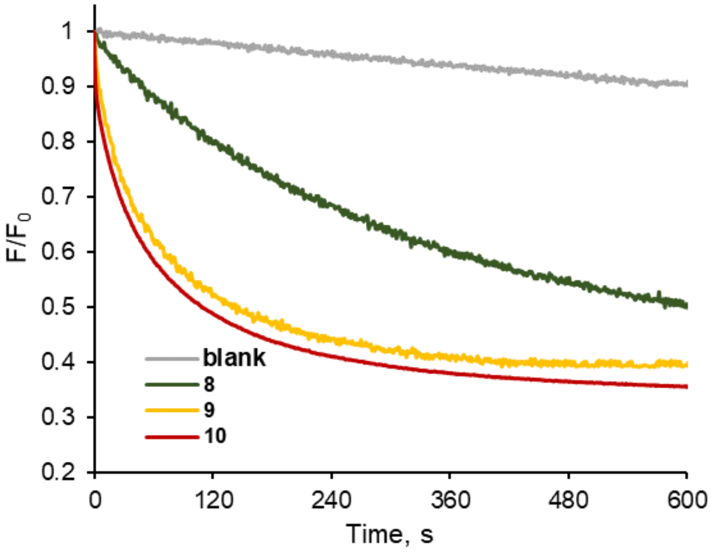

Supplementary Figure 8. Relative fluorescence F/F_0 for the transport of Cl^- into 200 nm LUVs by transporters **8-10** added externally as solution in DMF at 1:10 000 transporter:lipids ratio.

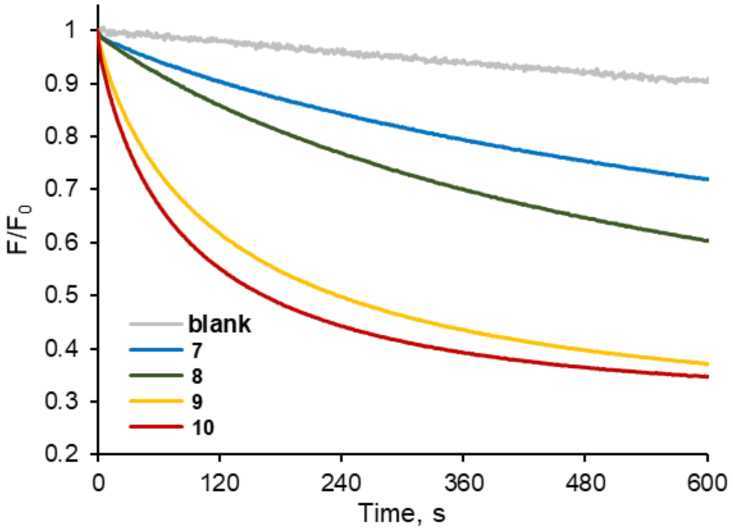

Supplementary Figure 9. Relative fluorescence F/F_0 for the transport of Cl^- into 200 nm LUVs by transporters **7-10** added externally as solution in DMF at 1:25 000 transporter:lipids ratio.

4. Data fitting of results from transporter preincorporation method

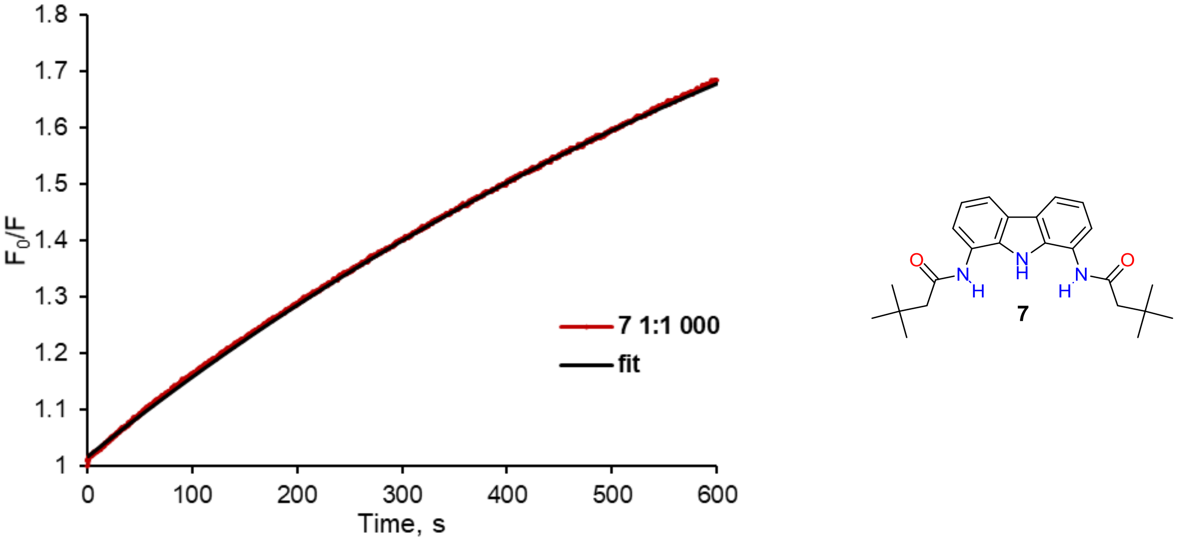

Supplementary Figure 10. Relative fluorescence F_0/F and single exponential fit for the transport of Cl^- into 200 nm LUVs by **7** preincorporated in the membrane (0.1 mol%).

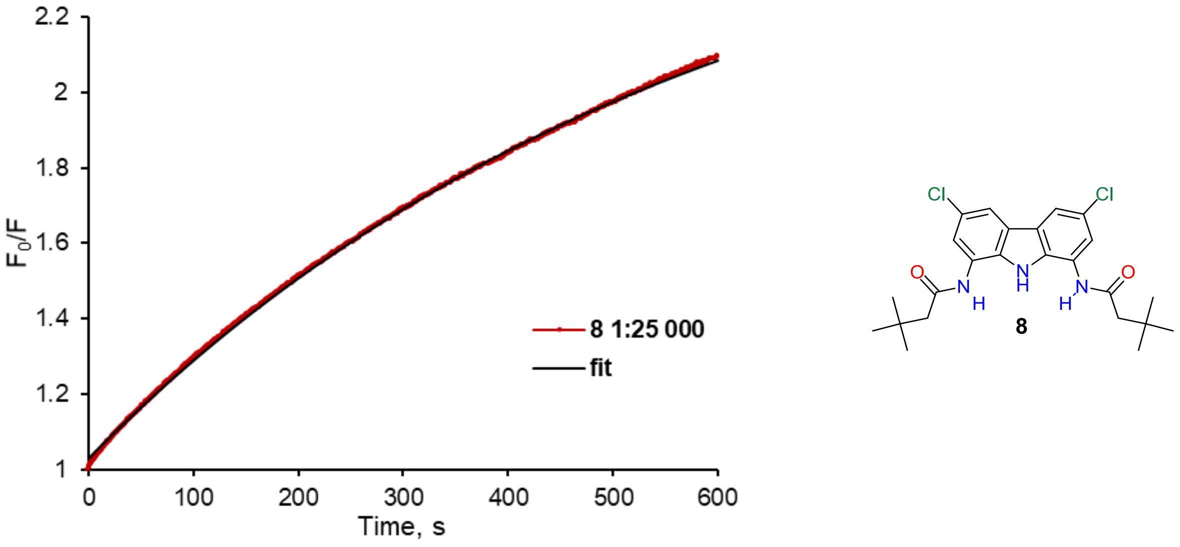

Supplementary Figure 11. Relative fluorescence F_0/F and single exponential fit for the transport of Cl^- into 200 nm LUVs by **8** preincorporated in the membrane (0.004 mol%).

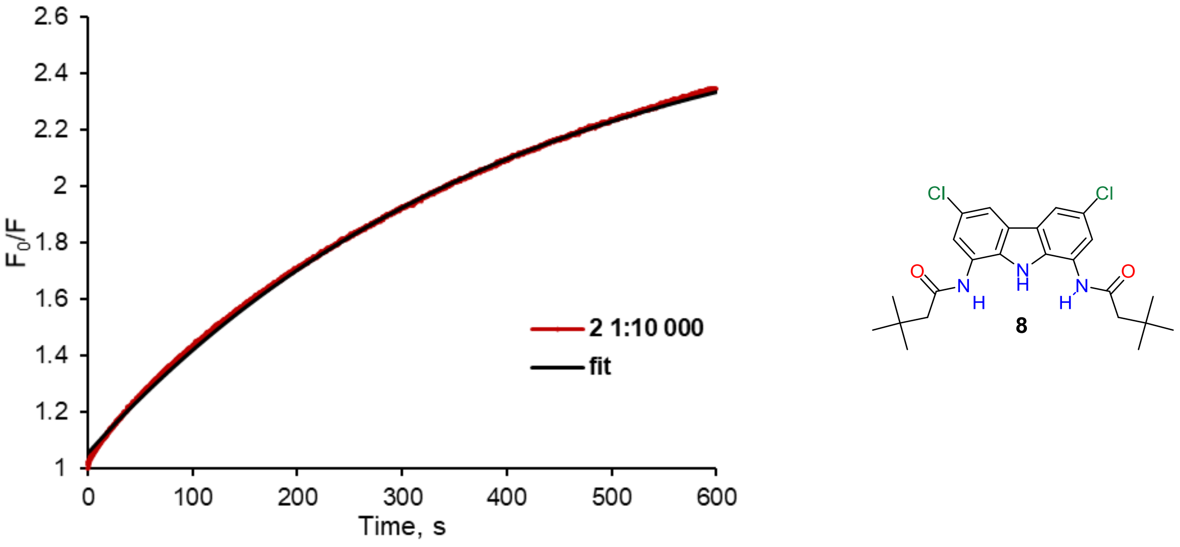

Supplementary Figure 12. Relative fluorescence F_0/F and single exponential fit for the transport of Cl^- into 200 nm LUVs by **8** preincorporated in the membrane (0.01 mol%).

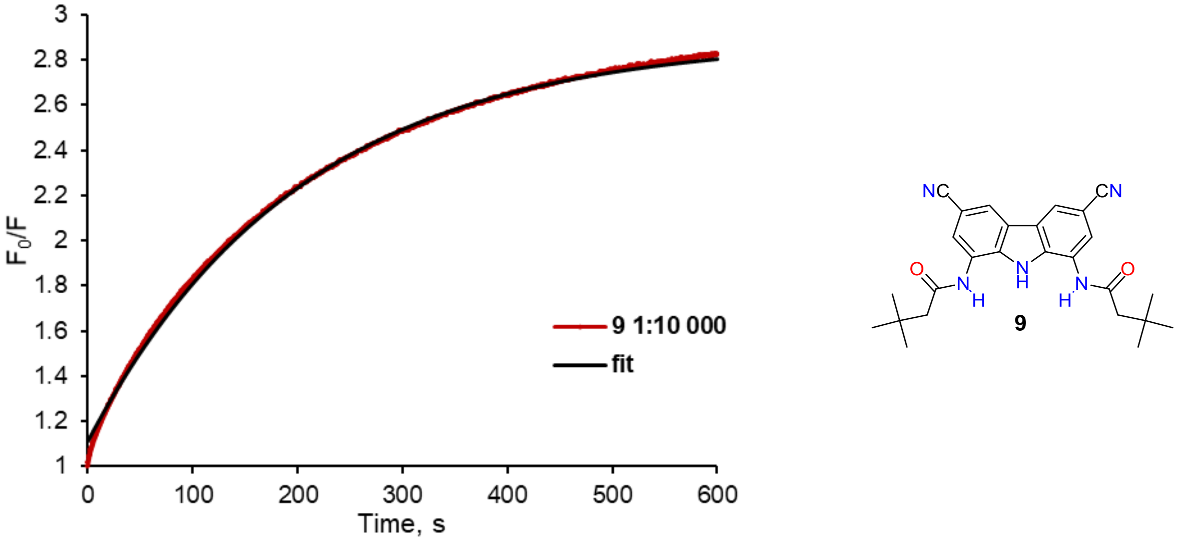

Supplementary Figure 13. Relative fluorescence F_0/F and single exponential fit for the transport of Cl^- into 200 nm LUVs by **9** preincorporated in the membrane (0.01 mol%).

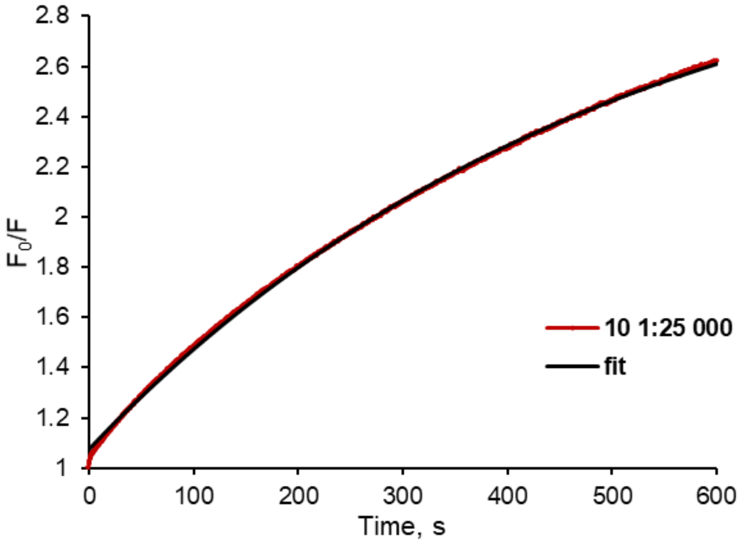

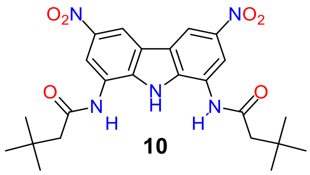

Supplementary Figure 14. Relative fluorescence F_0/F and single exponential fit for the transport of Cl^- into 200 nm LUVs by **10** preincorporated in the membrane (0.004 mol%).

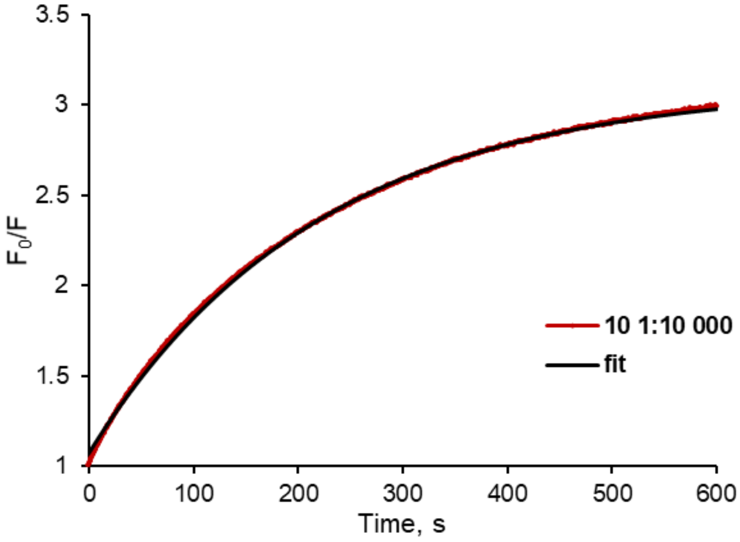

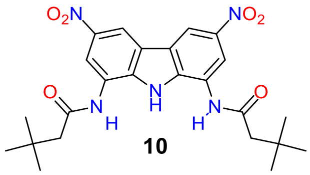

Supplementary Figure 15. Relative fluorescence F_0/F and single exponential fit for the transport of Cl^- into 200 nm LUVs by **10** preincorporated in the membrane (0.01 mol%).

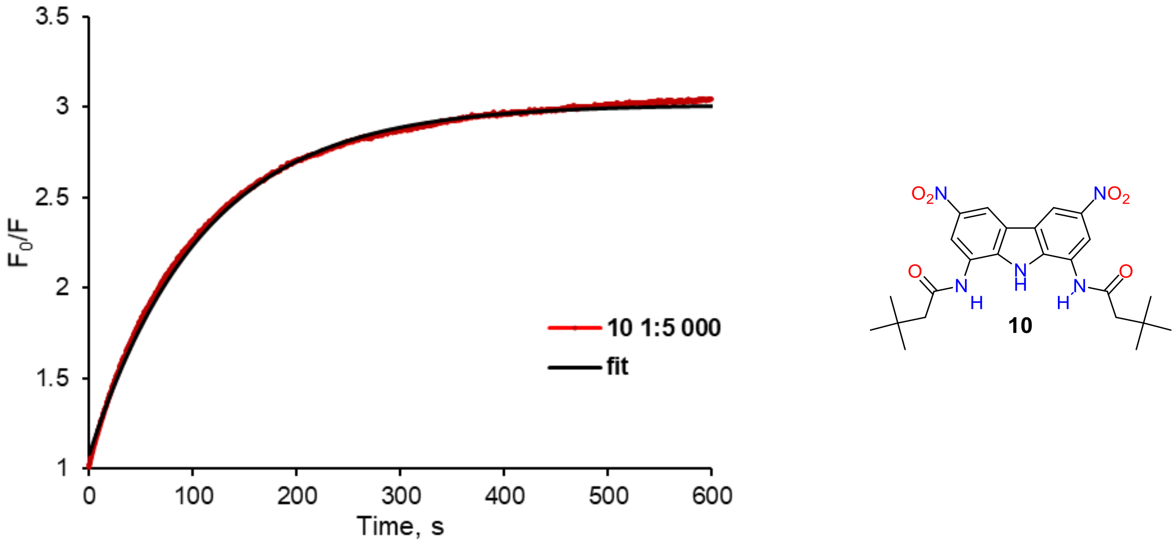

Supplementary Figure 16. Relative fluorescence F_0/F and single exponential fit for the transport of Cl^- into 200 nm LUVs by **10** preincorporated in the membrane (0.02 mol%).

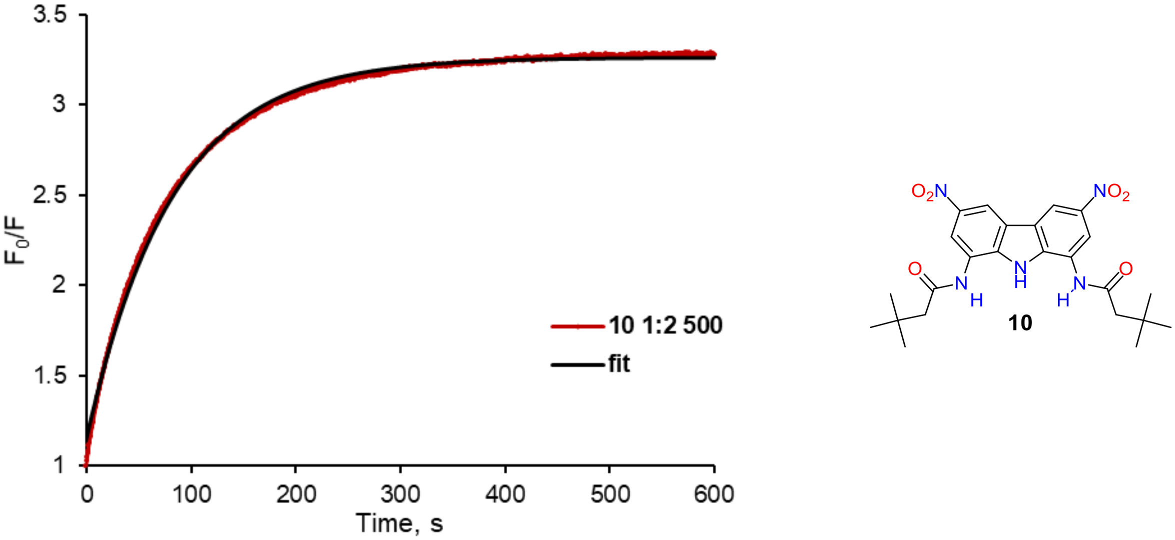

Supplementary Figure 17. Relative fluorescence F_0/F and single exponential fits for the transport of Cl^- into 200 nm LUVs by **10** preincorporated in the membrane (0.04 mol%).

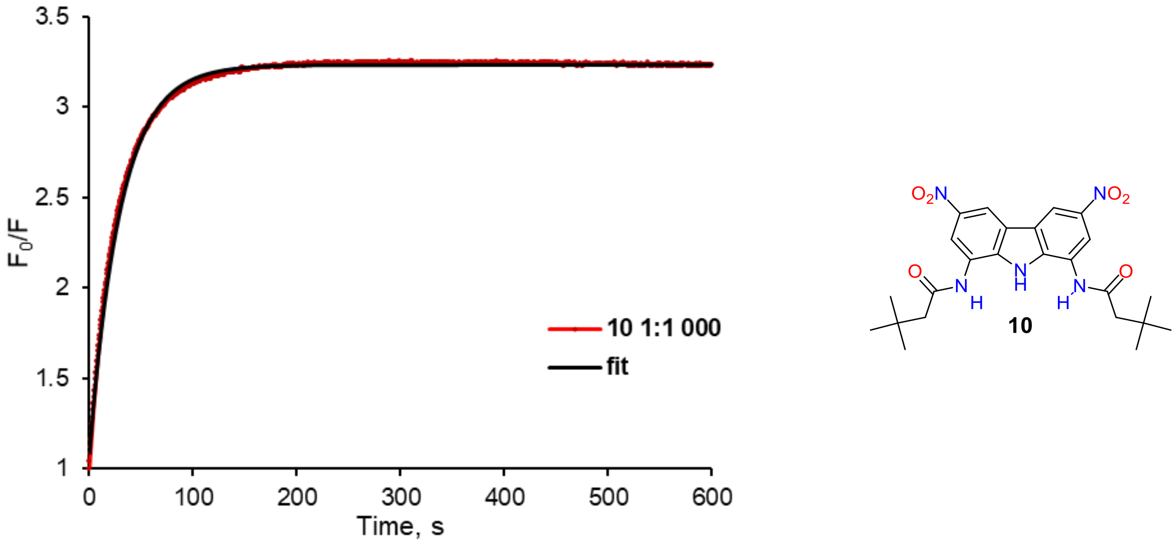

Supplementary Figure 18. Relative fluorescence F_0/F and single exponential fit for the transport of Cl^- into 200 nm LUVs by **10** preincorporated in the membrane (0.1 mol%).

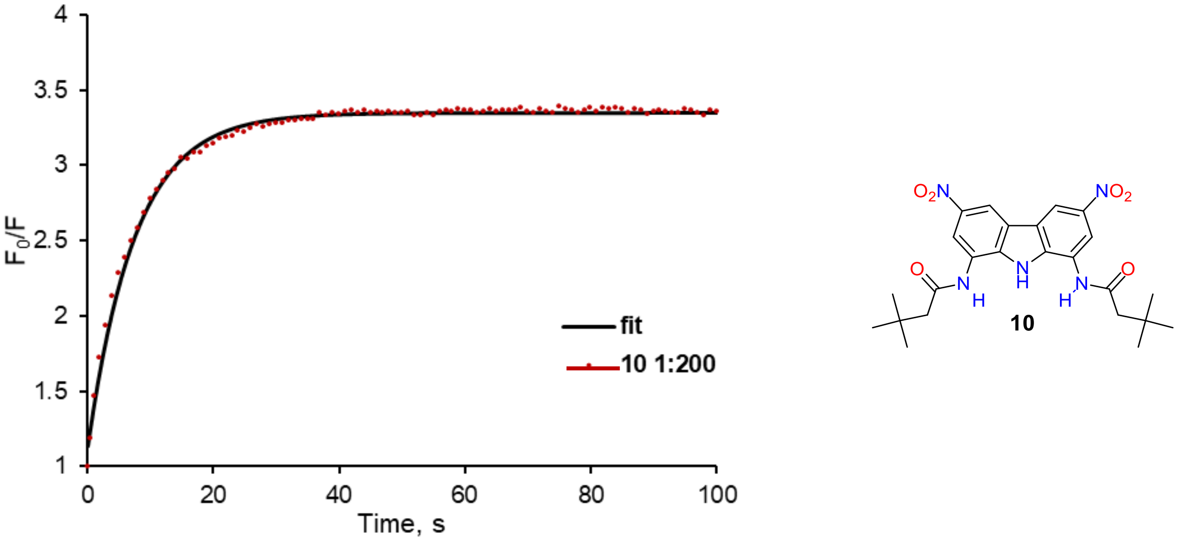

Supplementary Figure 19. Relative fluorescence F_0/F and single exponential fits for the transport of Cl^- into 200 nm LUVs by **10** preincorporated in the membrane (0.5 mol%).

5. Data fitting of results from externally added transporter method

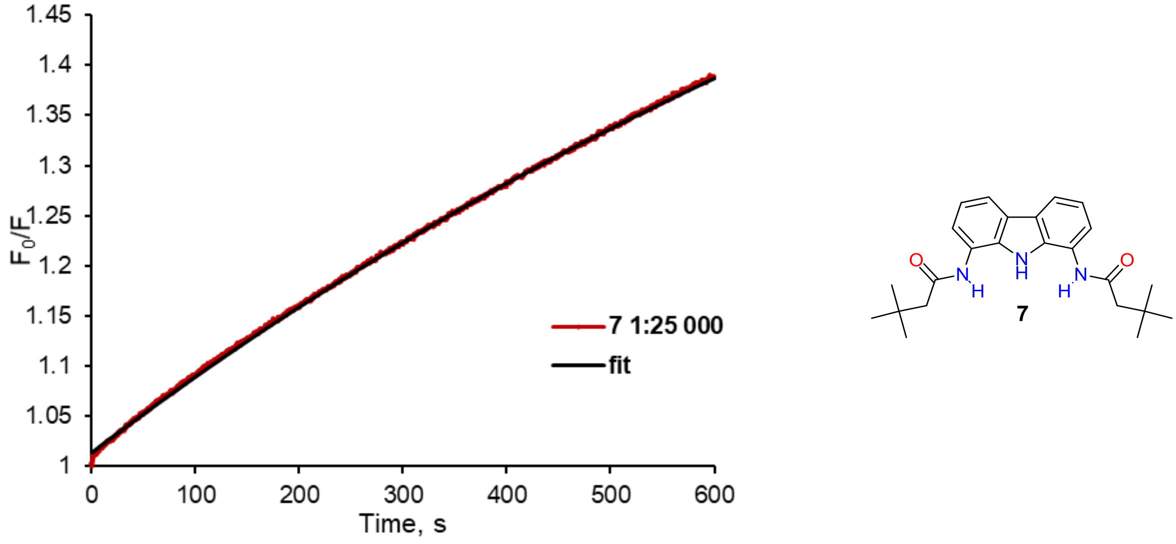

Supplementary Figure 20. Relative fluorescence F_0/F and single exponential fit for the transport of Cl^- into 200 nm LUVs by **7** added externally as solution in DMF (0.004 mol%).

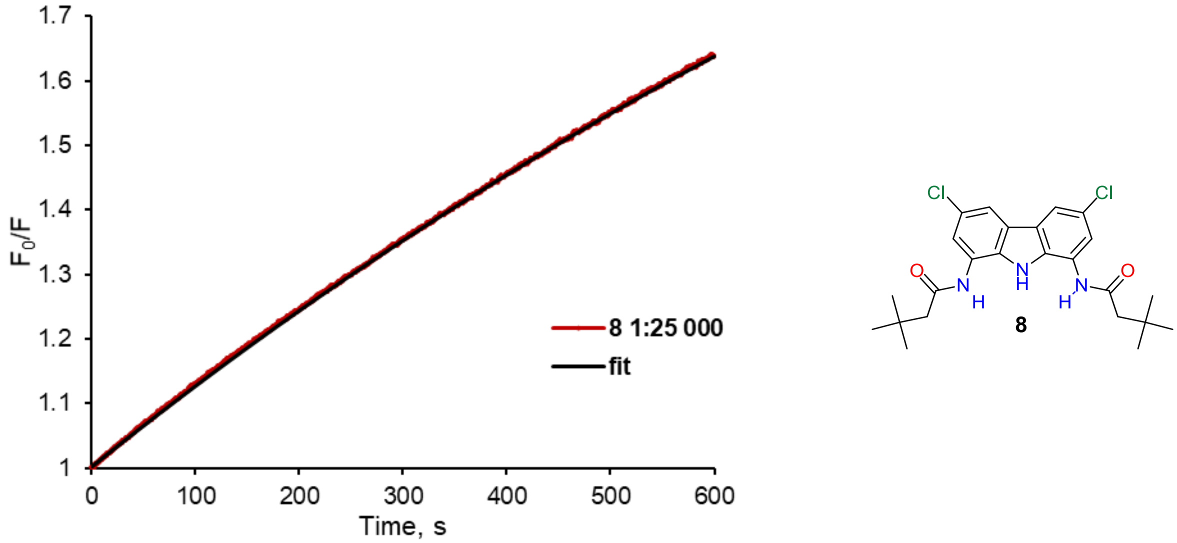

Supplementary Figure 21. Relative fluorescence F_0/F and single exponential fit for the transport of Cl^- into 200 nm LUVs by **8** added externally as solution in DMF (0.004 mol%).

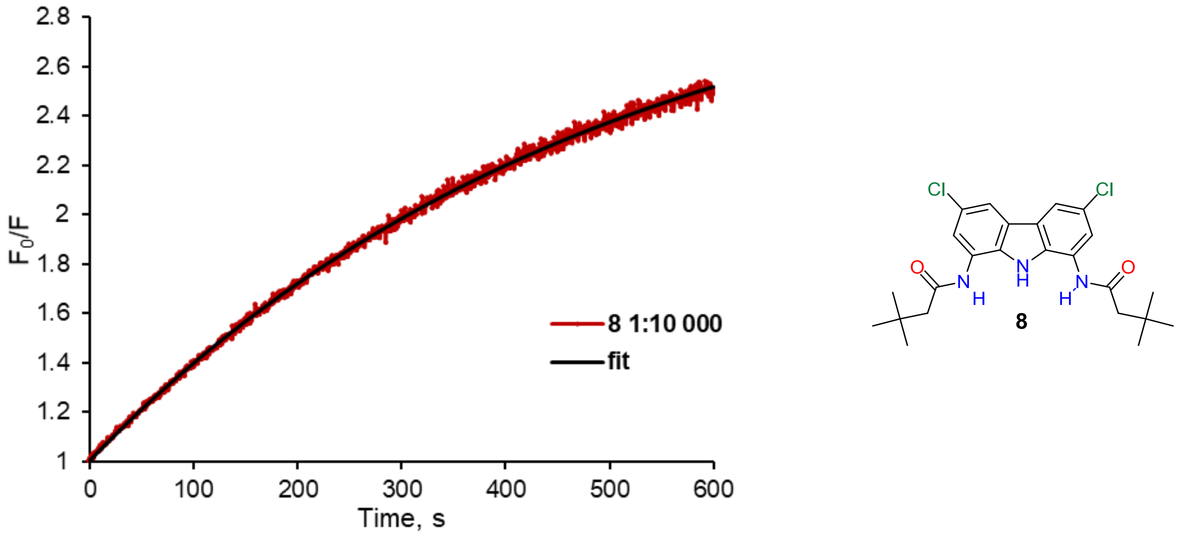

Supplementary Figure 22. Relative fluorescence *F_0/F* and single exponential fit for the transport of Cl^- into 200 nm LUVs by **8** added externally as solution in DMF (0.01 mol%).

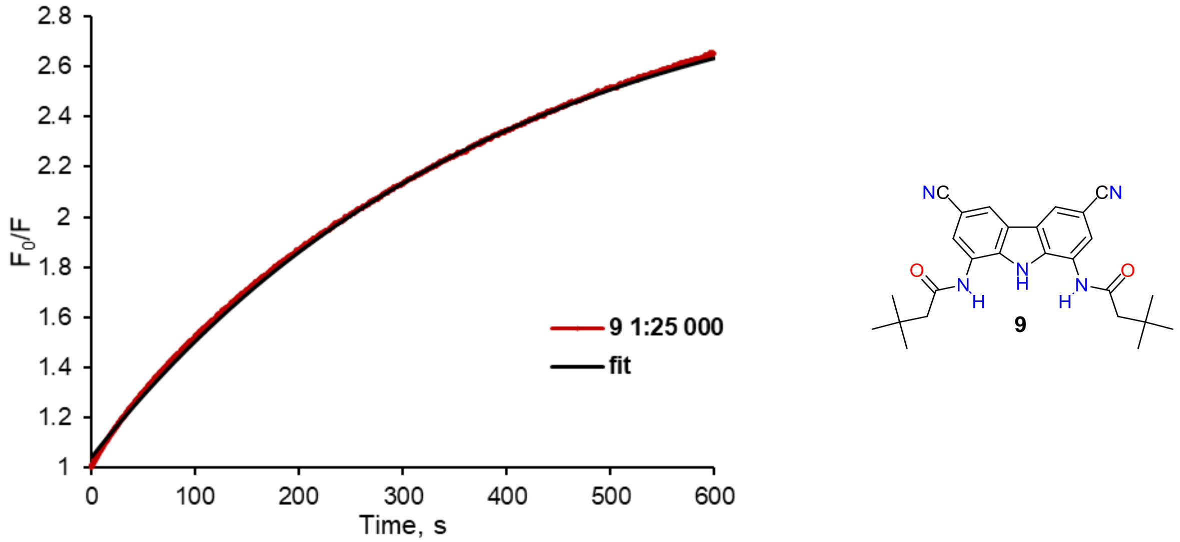

Supplementary Figure 23. Relative fluorescence *F_0/F* and single exponential fit for the transport of Cl^- into 200 nm LUVs by **9** added externally as solution in DMF (0.004 mol%).

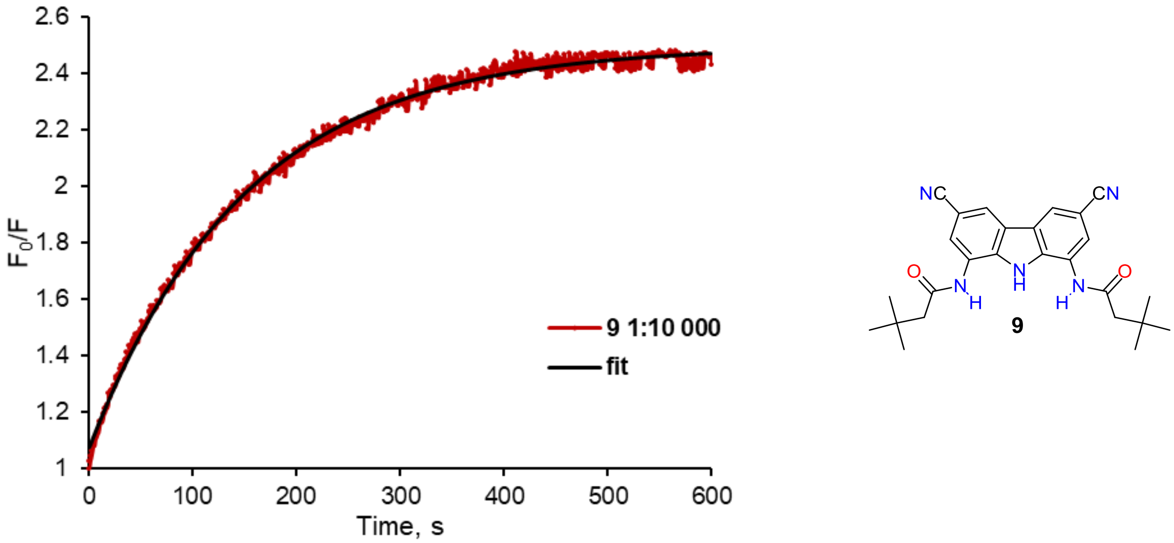

Supplementary Figure 24. Relative fluorescence F_0/F and single exponential fit for the transport of Cl^- into 200 nm LUVs by **9** added externally as solution in DMF (0.01 mol%).

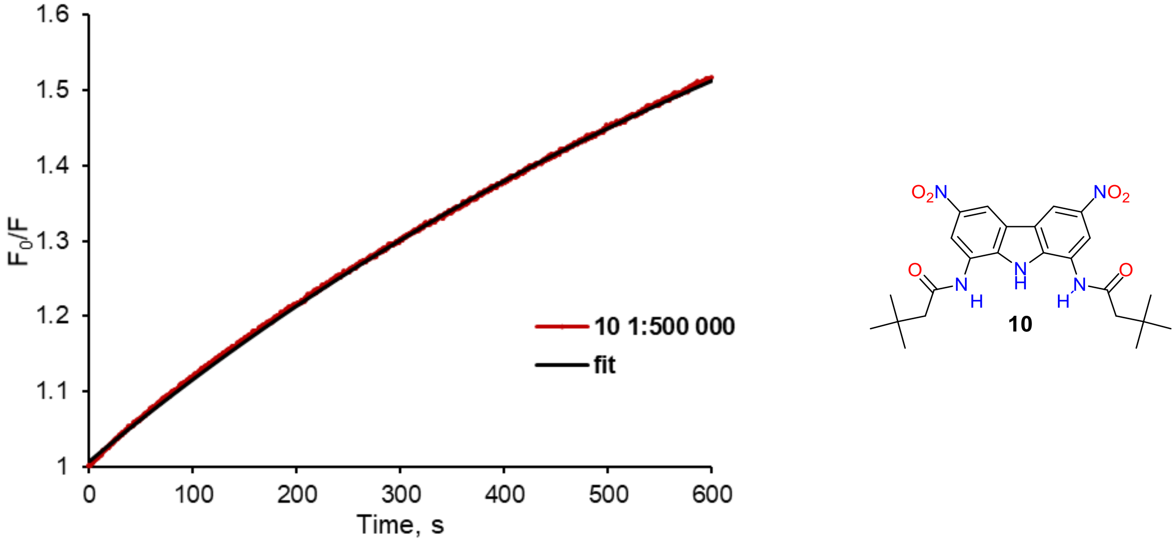

Supplementary Figure 25. Relative fluorescence F_0/F and single exponential fit for the transport of Cl^- into 200 nm LUVs by **10** added externally as solution in DMF (0.0002 mol%).

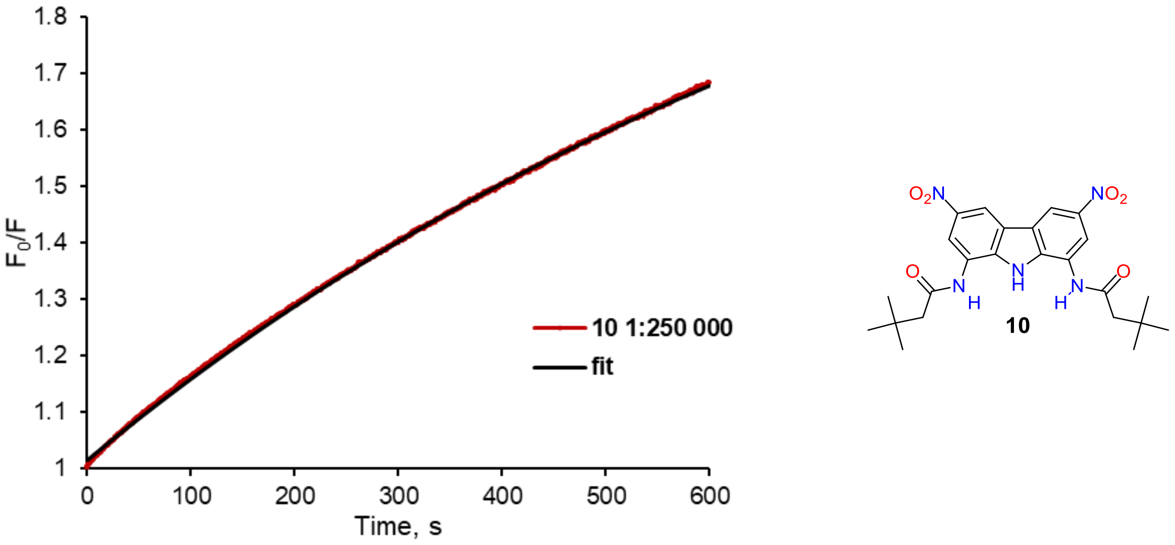

Supplementary Figure 26. Relative fluorescence F_0/F and single exponential fit for the transport of Cl^- into 200 nm LUVs by **10** added externally as solution in DMF (0.0004 mol%).

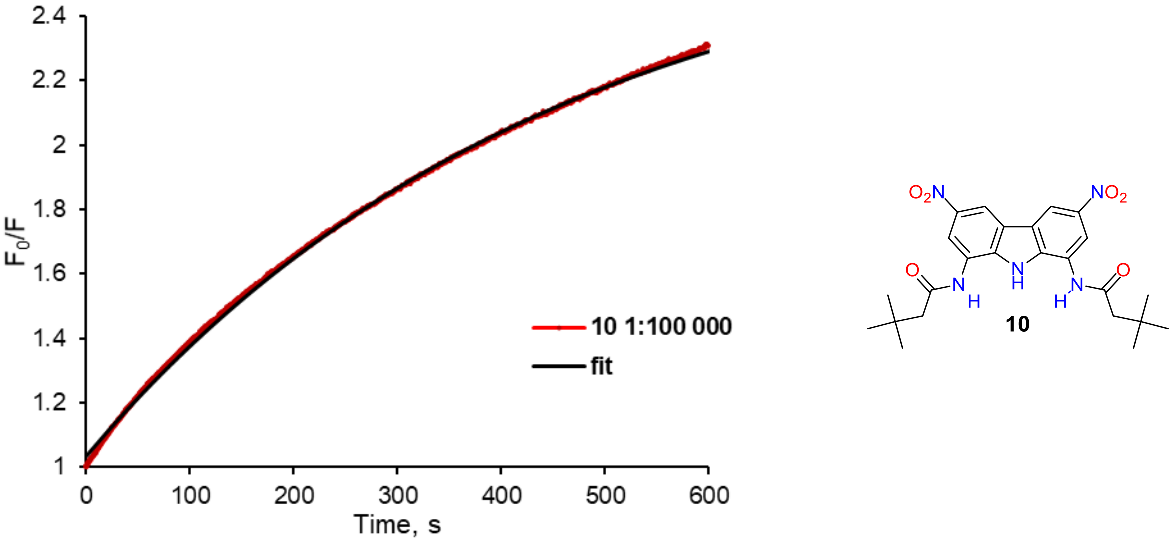

Supplementary Figure 27. Relative fluorescence F_0/F and single exponential fit for the transport of Cl^- into 200 nm LUVs by **10** added externally as solution in DMF (0.001 mol%).

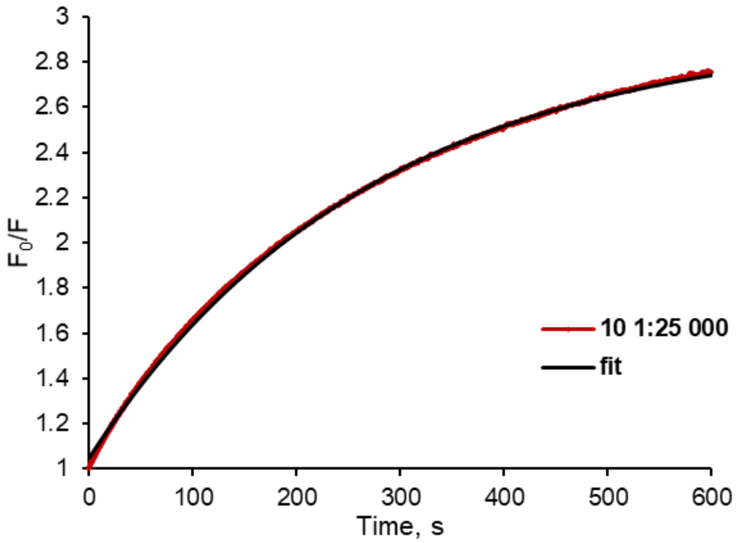

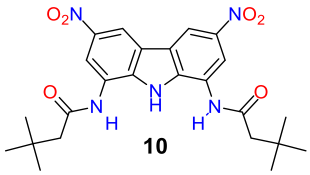

Supplementary Figure 28. Relative fluorescence F_0/F and single exponential fit for the transport of Cl^- into 200 nm LUVs by **10** added externally as solution in DMF (0.004 mol%).

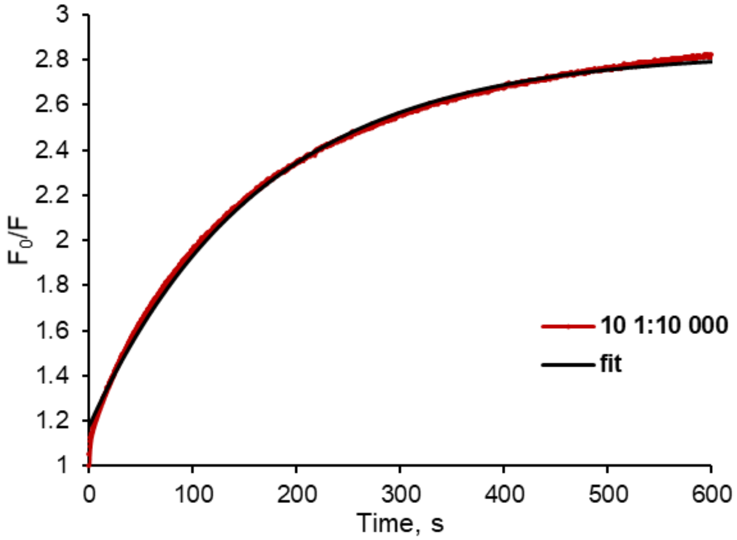

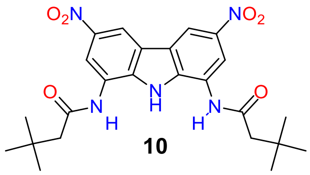

Supplementary Figure 29. Relative fluorescence F_0/F and single exponential fit for the transport of Cl^- into 200 nm LUVs by **10** added externally as solution in DMF (0.01 mol%).

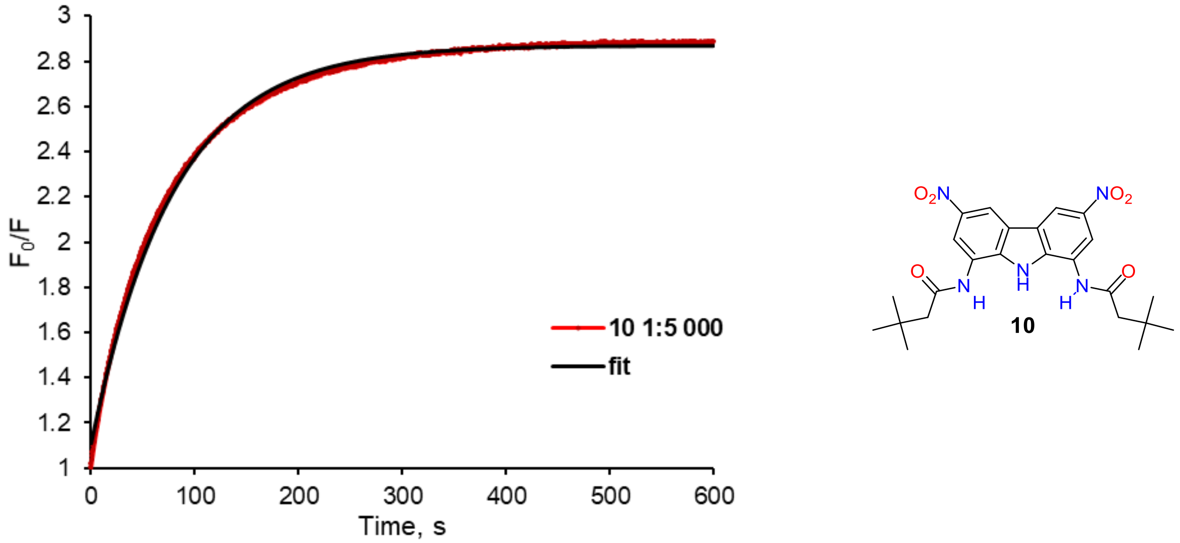

Supplementary Figure 30. Relative fluorescence F_0/F and single exponential fit for the transport of Cl^- into 200 nm LUVs by **10** added externally as solution in DMF (0.02 mol%).

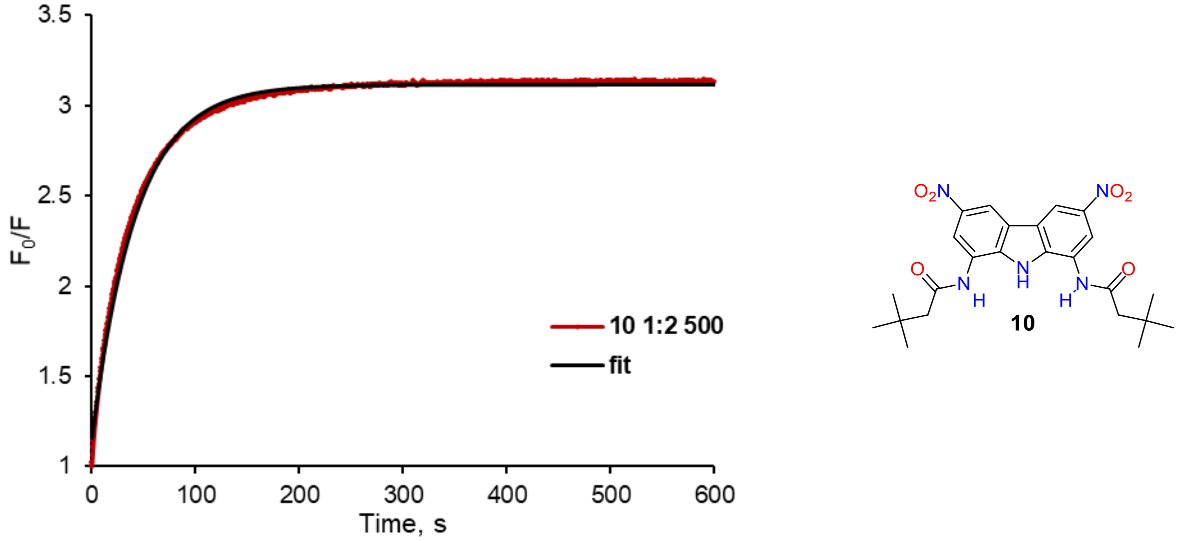

Supplementary Figure 31. Relative fluorescence F_0/F and single exponential fit for the transport of Cl^- into 200 nm LUVs by **10** added externally as solution in DMF (0.04 mol%).

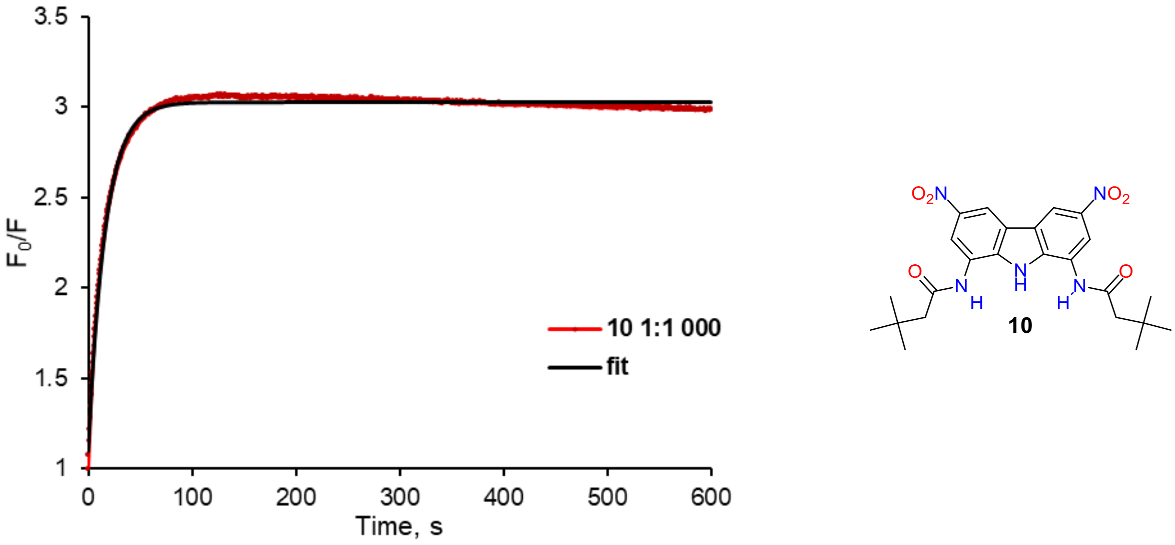

Supplementary Figure 32. Relative fluorescence F_0/F and single exponential fit for the transport of Cl^- into 200 nm LUVs by **10** added externally as solution in DMF (0.1 mol%).

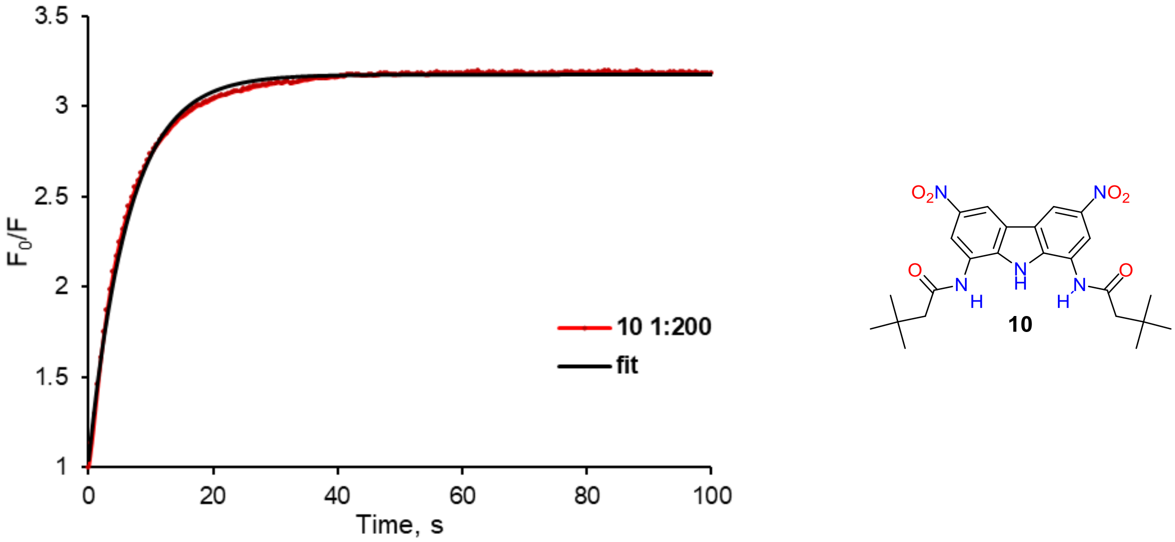

Supplementary Figure 33. Relative fluorescence F_0/F and single exponential fit for the transport of Cl^- into 200 nm LUVs by **10** added externally as solution in DMF (0.5 mol%).

6. Deliverability studies

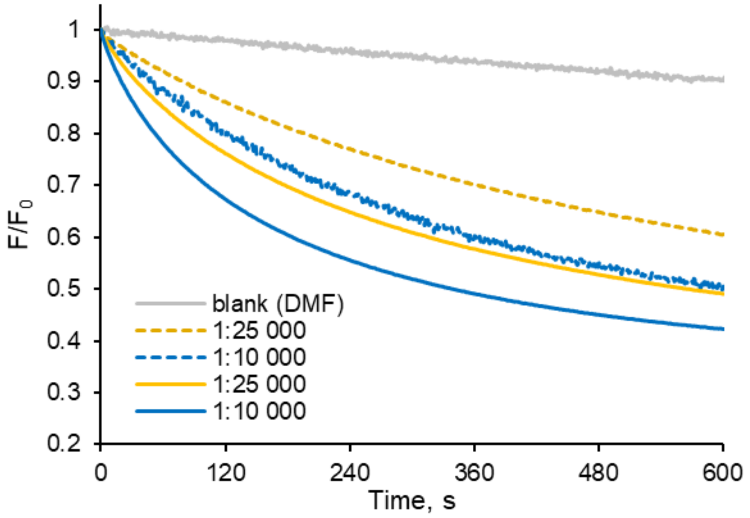

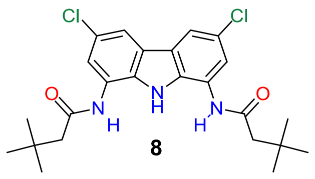

Supplementary Figure 34. Chloride transport into 200 nm LUVs mediated by **8** preincorporated in the membrane (solid line) or added externally as DMF solution (dashed line) in different anionophore-to-lipid ratios.

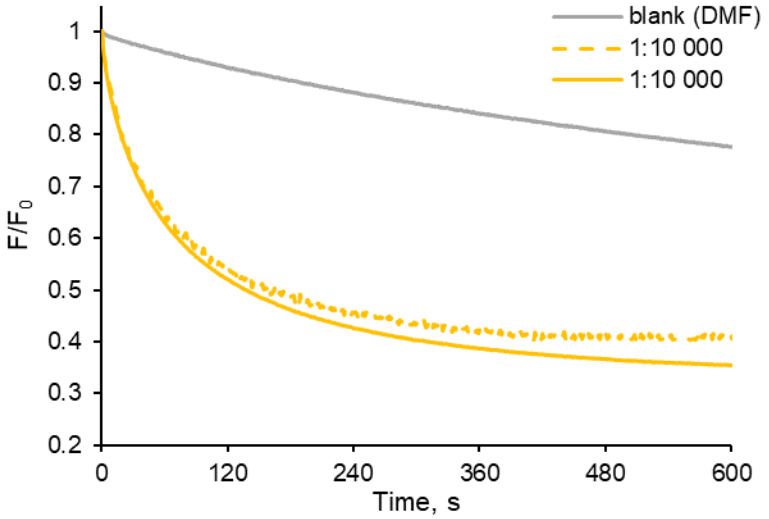

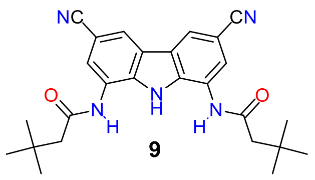

Supplementary Figure 35. Chloride transport into 200 nm LUVs mediated by **9** preincorporated in the membrane (solid line) or added externally as DMF solution (dashed line) in 1:10 000 anionophore-to-lipid ratio.

7. Hill analysis for receptor 10

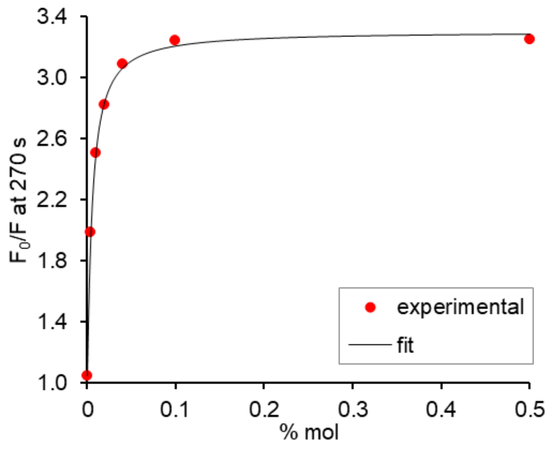

y = START + (END - START) * x^n / (k^n + x^n)	
Reduced Chi-Sqr	1.13E-03
Adj. R-Square	0.99827
START	1.047 ± 0
END	3.30231 ± 0.03158
EC50, 270	0.00559 ± 2.69577E-4
Hill coefficient	1.08413 ± 0.07069

Supplementary Figure 36. Plot of F_0/F (representative of intravesicular chloride concentration) 270 s after the addition of NaCl versus the concentration of **10** (preincorporated) inside the vesicles. The equation used for the fit for the calculation of $EC_{50,\ 270}$ and Hill coefficient is shown in the table.

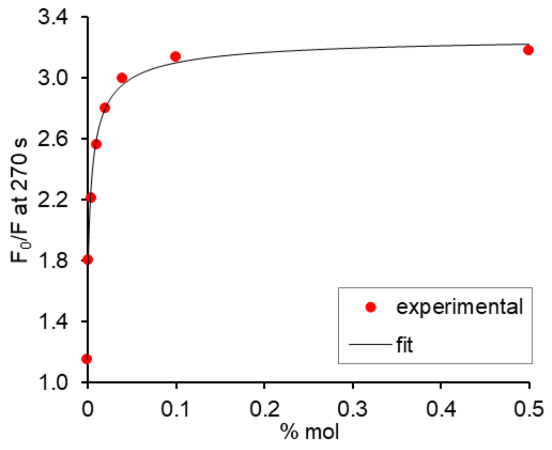

y = START + (END - START) * x^n / (k^n + x^n)	
Reduced Chi-Sqr	2.01E-03
Adj. R-Square	0.9961
START	1.15033 ± 0
END	3.28978 ± 0.05477
EC50, 270	0.00365 ± 3.96842E-4
Hill coefficient	0.71028 ± 0.05739

Supplementary Figure 37. Plot of F_0/F (representative of intravesicular chloride concentration) 270 s after the addition of NaCl versus the concentration of **10** (added externally as solution in DMF) inside the vesicles. The equation used for the fit for the calculation of $EC_{50,\ 270}$ and Hill coefficient is shown in the table.

8. Quantification of pH-dependent chloride transport rates

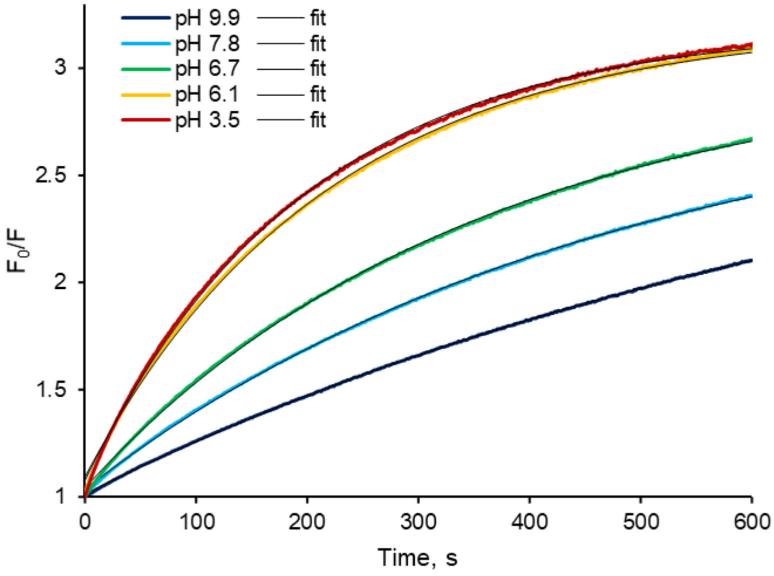

pH	$t_{1/2}$, s	I, s^{-1}
3.5	138	0.01330
6.1	163	0.01298
6.7	242	0.00564
7.8	327	0.00526
9.9	537	0.00333

Supplementary Figure 38. Relative fluorescence F_0/F and single exponential fits for the pH-dependent Cl^- transport into 200 nm LUVs with preincorporated **10** at 1:10 000 transporter:lipids ratio. Quantification of pH-dependent transport rates was accomplished by fitting the F_0/F curve (0-600 s) to a single exponential decay function (for half-life time value, $t_{1/2}$) and to a double exponential decay function (for initial rate, *I*).

The obtained data were fitted to a double exponential decay function and the initial rate *I* of chloride transport was calculated and plotted as a function of pH. The equation used for the fit for the calculation of pKa is shown in the table below:

Model	pKa (User)
y = MAX + ((MIN - MAX) / (1 + 10 ^ (pKa - x)))	
Reduced Chi-Sqr	2.40E-06
Adj. R-Square	0.87175
MAX	0.01368 ± 0.00152
MIN	0.00383 ± 0.00115
pKa	6.37843 ± 0.29741

9. Crystal data and structure refinement

Table S1. Crystal data and structure refinement for **9**×Cl$^-$ complex.

**Identification code**	**K1**
**Empirical formula**	$C_{42}H_{65}ClN_6O_2$
**Formula weight**	721.45
**Temperature/K**	200.00(10)
**Crystal system**	monoclinic
**Space group**	P2_1
***a*/Å**	13.7154(7)
***b*/Å**	16.2713(7)
***c*/Å**	19.0787(10)
***α*/°**	90
***β*/°**	92.053(5)
***γ*/°**	90
**Volume/Å^3**	4255.0(4)
Z	4
**ρ_{calc} g/cm^3**	1.126
***μ*/mm^{-1}**	0.130
***F*(000)**	1568.0
**Crystal size/mm^3**	0.934 × 0.286 × 0.156
**Radiation**	MoKα (λ = 0.71073)
**2*Θ* range for data collection/°**	4.486 to 52.744
**Index ranges**	$-17 \le h \le 17$, $-20 \le k \le 19$, $-23 \le l \le 23$
**Reflections collected**	72830
**Independent reflections**	17319 [R_{int} = 0.0651, R_{sigma} = 0.0616]
**Data/restraints/parameters**	17319/1/939
**Goodness-of-fit on F^2**	1.081
**Final *R* indexes [*I*>=2*σ* (*I*)]**	R_1 = 0.0960, wR_2 = 0.2435
**Final *R* indexes [all data]**	R_1 = 0.1172, wR_2 = 0.2566
**Largest diff. peak/hole / e Å^{-3}**	0.61/-0.37
**Flack parameter**	0.16(3)

Table S2. Bond lengths for **9**×Cl^- complex.

Atom	Atom	Length/Å	Atom	Atom	Length/Å
**N1C**	C2C	1.369(10)	C6B	C7B	1.369(11)
**N1C**	C2D	1.369(10)	C7A	N8A	1.416(10)
**C2C**	C3C	1.412(10)	C7B	N8B	1.416(10)
**C2C**	C7C	1.399(11)	N8A	C9A	1.343(10)
**C2D**	C3D	1.415(11)	N8B	C9B	1.351(10)
**C2D**	C7D	1.421(11)	C9A	C10A	1.497(11)
**C3C**	C3D	1.436(10)	C9A	O17A	1.246(9)
**C3C**	C4C	1.403(10)	C9B	C10B	1.507(12)
**C3D**	C4D	1.409(11)	C9B	O17B	1.219(10)
**C4C**	C5C	1.408(11)	C10A	C11A	1.562(11)
**C4D**	C5D	1.383(11)	C10B	C11B	1.560(12)
**C5C**	C6C	1.390(11)	C11A	C12A	1.519(13)
**C5C**	C15C	1.443(11)	C11A	C13A	1.527(13)
**C5D**	C6D	1.413(11)	C11A	C14A	1.534(12)
**C5D**	C15D	1.446(11)	C11B	C12B	1.520(14)
**C6C**	C7C	1.397(11)	C11B	C13B	1.542(17)
**C6D**	C7D	1.364(11)	C11B	C14B	1.516(14)
**C7C**	N8C	1.426(10)	C15A	N16A	1.155(11)
**C7D**	N8D	1.410(10)	C15B	N16B	1.134(12)
**N8C**	C9C	1.358(10)	N19A	C20A	1.525(9)
**N8D**	C9D	1.367(11)	N19A	C24A	1.532(10)
**C9C**	C10C	1.511(12)	N19A	C28A	1.516(10)
**C9C**	O17C	1.229(10)	N19A	C32A	1.529(10)
**C9D**	C10D	1.515(13)	C20A	C21A	1.500(12)
**C9D**	O17D	1.223(11)	C21A	C22A	1.554(12)
**C10C**	C11C	1.519(12)	C22A	C23A	1.524(14)
**C10D**	C11D	1.497(12)	C24A	C25A	1.523(12)
**C11C**	C12C	1.509(18)	C25A	C26A	1.511(14)
**C11C**	C13C	1.506(15)	C26A	C27A	1.547(16)
**C11C**	C14C	1.54(2)	C28A	C29A	1.537(11)
**C11D**	C12D	1.493(15)	C29A	C30A	1.518(14)
**C11D**	C13D	1.521(17)	C30A	C31A	1.513(16)
**C11D**	C14D	1.533(17)	C32A	C33A	1.496(12)
**C15C**	N16C	1.147(11)	C33A	C34A	1.531(13)
**C15D**	N16D	1.132(11)	C34A	C35A	1.499(15)
**N1A**	C2A	1.386(9)	N19B	C20B	1.504(12)
**N1A**	C2B	1.366(9)	N19B	C24B	1.532(12)
**C2A**	C3A	1.417(10)	N19B	C28B	1.546(12)
**C2A**	C7A	1.408(11)	N19B	C32B	1.518(11)
**C2B**	C3B	1.427(10)	C20B	C21B	1.506(16)
**C2B**	C7B	1.407(10)	C21B	C22B	1.582(19)
**C3A**	C3B	1.444(10)	C22B	C23B	1.49(2)
**C3A**	C4A	1.399(10)	C24B	C25B	1.508(15)
**C3B**	C4B	1.375(10)	C25B	C26B	1.509(15)
**C4A**	C5A	1.375(11)	C26B	C27B	1.48(2)
**C4B**	C5B	1.415(11)	C28B	C29B	1.511(15)
**C5A**	C6A	1.397(11)	C29B	C30B	1.512(15)
**C5A**	C15A	1.450(11)	C30B	C31B	1.498(16)
**C5B**	C6B	1.392(11)	C32B	C33B	1.507(14)
**C5B**	C15B	1.443(12)	C33B	C34B	1.531(13)
**C6A**	C7A	1.366(11)	C34B	C35B	1.497(16)

Table S3. Valence angles for **9**×Cl^- complex.

Atom	Atom	Atom	Angle/°	Atom	Atom	Atom	Angle/°
C2D	N1C	C2C	*109.1(6)*	C4B	C5B	C15B	*118.2(7)*
N1C	C2C	C3C	*109.3(6)*	C6B	C5B	C4B	*121.0(7)*
N1C	C2C	C7C	*128.1(7)*	C6B	C5B	C15B	*120.8(7)*
C7C	C2C	C3C	*122.5(7)*	C7A	C6A	C5A	*122.5(7)*
N1C	C2D	C3D	*109.0(7)*	C7B	C6B	C5B	*123.0(7)*
N1C	C2D	C7D	*127.8(7)*	C2A	C7A	N8A	*119.0(6)*
C3D	C2D	C7D	*123.2(7)*	C6A	C7A	C2A	*116.6(7)*
C2C	C3C	C3D	*106.2(6)*	C6A	C7A	N8A	*124.4(7)*
C4C	C3C	C2C	*119.7(7)*	C2B	C7B	N8B	*122.2(7)*
C4C	C3C	C3D	*134.0(6)*	C6B	C7B	C2B	*116.4(7)*
C2D	C3D	C3C	*106.4(6)*	C6B	C7B	N8B	*121.4(7)*
C4D	C3D	C2D	*118.8(7)*	C9A	N8A	C7A	*126.6(6)*
C4D	C3D	C3C	*134.3(7)*	C9B	N8B	C7B	*123.9(7)*
C3C	C4C	C5C	*117.4(7)*	N8A	C9A	C10A	*116.0(6)*
C5D	C4D	C3D	*117.4(7)*	O17A	C9A	N8A	*122.1(7)*
C4C	C5C	C15C	*118.8(7)*	O17A	C9A	C10A	*121.9(7)*
C6C	C5C	C4C	*122.1(7)*	N8B	C9B	C10B	*115.7(7)*
C6C	C5C	C15C	*119.1(7)*	O17B	C9B	N8B	*120.8(8)*
C4D	C5D	C6D	*122.5(7)*	O17B	C9B	C10B	*123.5(8)*
C4D	C5D	C15D	*119.6(7)*	C9A	C10A	C11A	*113.7(7)*
C6D	C5D	C15D	*117.7(7)*	C9B	C10B	C11B	*115.4(7)*
C5C	C6C	C7C	*121.2(7)*	C12A	C11A	C10A	*111.7(8)*
C7D	C6D	C5D	*121.9(7)*	C12A	C11A	C13A	*109.9(8)*
C2C	C7C	N8C	*117.9(7)*	C12A	C11A	C14A	*109.7(8)*
C6C	C7C	C2C	*116.9(7)*	C13A	C11A	C10A	*107.8(7)*
C6C	C7C	N8C	*125.2(7)*	C13A	C11A	C14A	*107.9(8)*
C6D	C7D	C2D	*115.7(7)*	C14A	C11A	C10A	*109.9(7)*
C6D	C7D	N8D	*126.3(7)*	C12B	C11B	C10B	*111.0(7)*
N8D	C7D	C2D	*118.0(7)*	C12B	C11B	C13B	*106.8(11)*
C9C	N8C	C7C	*125.8(7)*	C13B	C11B	C10B	*107.1(8)*
C9D	N8D	C7D	*127.7(7)*	C14B	C11B	C10B	*111.4(8)*
N8C	C9C	C10C	*114.8(8)*	C14B	C11B	C12B	*107.2(9)*
O17C	C9C	N8C	*123.3(8)*	C14B	C11B	C13B	*113.3(11)*
O17C	C9C	C10C	*121.9(8)*	N16A	C15A	C5A	*178.6(10)*
N8D	C9D	C10D	*114.7(7)*	N16B	C15B	C5B	*179.0(11)*
O17D	C9D	N8D	*121.2(8)*	C20A	N19A	C24A	*106.7(6)*
O17D	C9D	C10D	*124.1(8)*	C20A	N19A	C32A	*111.1(6)*
C9C	C10C	C11C	*114.9(8)*	C28A	N19A	C20A	*111.1(6)*
C11D	C10D	C9D	*118.8(8)*	C28A	N19A	C24A	*111.1(6)*
C10C	C11C	C14C	*108.2(10)*	C28A	N19A	C32A	*106.9(5)*
C12C	C11C	C10C	*109.6(10)*	C32A	N19A	C24A	*110.1(6)*
C12C	C11C	C14C	*110.5(15)*	C21A	C20A	N19A	*115.6(6)*
C13C	C11C	C10C	*110.0(8)*	C20A	C21A	C22A	*109.4(7)*
C13C	C11C	C12C	*108.5(11)*	C23A	C22A	C21A	*112.6(9)*
C13C	C11C	C14C	*110.1(12)*	C25A	C24A	N19A	*116.3(7)*
C10D	C11D	C13D	*106.9(9)*	C26A	C25A	C24A	*110.4(8)*
C10D	C11D	C14D	*109.7(9)*	C25A	C26A	C27A	*110.4(9)*
C12D	C11D	C10D	*115.0(10)*	N19A	C28A	C29A	*115.4(6)*
C12D	C11D	C13D	*108.5(10)*	C30A	C29A	C28A	*109.4(7)*

C12D	C11D	C14D	*109.4(12)*	C31A	C30A	C29A	*114.9(9)*
C13D	C11D	C14D	*107.0(12)*	C33A	C32A	N19A	*116.6(7)*
N16C	C15C	C5C	*178.8(10)*	C32A	C33A	C34A	*112.5(7)*
N16D	C15D	C5D	*177.2(10)*	C35A	C34A	C33A	*115.6(9)*
C2B	N1A	C2A	*109.4(6)*	C20B	N19B	C24B	*111.4(7)*
N1A	C2A	C3A	*109.1(6)*	C20B	N19B	C28B	*110.5(8)*
N1A	C2A	C7A	*129.5(7)*	C20B	N19B	C32B	*106.8(6)*
C7A	C2A	C3A	*121.3(6)*	C24B	N19B	C28B	*107.4(7)*
N1A	C2B	C3B	*108.8(6)*	C32B	N19B	C24B	*110.3(7)*
N1A	C2B	C7B	*129.7(7)*	C32B	N19B	C28B	*110.6(7)*
C7B	C2B	C3B	*121.5(6)*	N19B	C20B	C21B	*117.0(8)*
C2A	C3A	C3B	*106.1(6)*	C20B	C21B	C22B	*113.7(10)*
C4A	C3A	C2A	*120.3(7)*	C23B	C22B	C21B	*100.4(18)*
C4A	C3A	C3B	*133.5(7)*	C25B	C24B	N19B	*115.8(8)*
C2B	C3B	C3A	*106.6(6)*	C24B	C25B	C26B	*111.6(10)*
C4B	C3B	C2B	*120.8(6)*	C27B	C26B	C25B	*115.3(14)*
C4B	C3B	C3A	*132.6(7)*	C29B	C28B	N19B	*114.3(8)*
C5A	C4A	C3A	*117.4(7)*	C28B	C29B	C30B	*112.2(9)*
C3B	C4B	C5B	*117.2(7)*	C31B	C30B	C29B	*113.0(10)*
C4A	C5A	C6A	*121.8(7)*	C33B	C32B	N19B	*115.5(7)*
C4A	C5A	C15A	*119.2(7)*	C32B	C33B	C34B	*110.2(8)*
C6A	C5A	C15A	*118.9(7)*	C35B	C34B	C33B	*114.3(9)*
